# The prefrontal cortex controls memory organization in the hippocampus

**DOI:** 10.1038/s41593-026-02231-1

**Published:** 2026-04-28

**Authors:** André F. de Sousa, Zachary E. Zeidler, Daniel G. Almeida-Filho, Yang Shen, Alessandro Luchetti, Shana Simanian, Mouaz Mardini, Laura A. DeNardo, Alcino J. Silva

**Affiliations:** 1 Departments of Neurobiology, Psychiatry & Biobehavioral Sciences, and Psychology, Integrative Center for Learning and Memory, Brain Research Institute, UCLA, Los Angeles, CA USA; 2 Departments of Physiology and Neurobiology, UCLA, Los Angeles, CA USA; 3 SENAI Institute of Innovation in Advanced Health Systems, University Center SENAI, CIMATEC, Salvador, Bahia Brazil; 4 Present Address: Bioscience and Biomedical Engineering Thrust, Brain and Intelligence Research Institute, The Hong Kong University of Science and Technology (Guangzhou), Guangzhou, China; 5 Present Address: Program in Neurosciences & Mental Health, Hospital for Sick Children, Toronto, Ontario Canada

**Keywords:** Hippocampus, Neural circuits

## Abstract

Prior memories can be integrated with novel experiences during learning to facilitate memory organization. This process must be tightly regulated to prevent inappropriate integration of unrelated memories. However, the biological mechanisms underlying such control are currently unknown. Using multiple imaging, chemogenetic and optogenetic techniques in mice, we demonstrate that the ventromedial prefrontal cortex is recruited over time to control memory integration in the hippocampus according to contextual similarities between experiences. This control is achieved through direct projections to the medial entorhinal cortex that modulate entorhinal activity, ensemble overlap in the dorsal hippocampus, memory linking, activity of neurogliaform cells in the dorsal CA1 and memory allocation. Together, our results provide new insights into the mechanisms controlling crucial processes of memory organization in the mammalian brain.

## Abstract (web summary)

Related memories are sometimes encoded in overlapping neurons. The authors show that the prefrontal cortex controls this type of memory organization in the hippocampus through direct projections to the medial entorhinal cortex.

## Main

Memories are not formed in isolation and previous knowledge often influences how novel information is acquired and organized^1^. Memory organization is influenced by different dimensions of experience including the temporal and spatial contexts in which memories are encoded, as well as the existence of perceptual and conceptual similarities between memories^2–4^. When two memories share these dimensions, they tend to be encoded in many of the same neurons such that retrieving one memory increases the likelihood of retrieving the other, a process known as memory integration^2,3^. This process must be tightly regulated to prevent inappropriate integration of unrelated memories, which can lead to the formation of false associations often observed in many psychiatric disorders^5–7^. Nonetheless, the biological mechanisms that modulate the integration or separation of memories remain poorly understood. In particular, little is known about the processes that control the organization of memories acquired several days apart, a timeframe that allows for long-term memories to influence novel encoding.

The prefrontal cortex (PFC) is thought to store and process long-term memories^8^ and several studies have suggested that interactions between this brain region and the hippocampus (HPC) may be important for the organization of memories in the context of prior knowledge^9–12^. However, the biological mechanisms and neuronal pathways supporting this process remain poorly understood.

Here, we demonstrate that, in mice, the ventromedial PFC (vmPFC) can directly influence memory organization by controlling whether two memories are integrated or separated in the HPC. This top-down modulation of memory integration depends on whether both episodes share the same spatial context and is evident when memories are encoded 7 days but not 5 hours apart, suggesting a gradual involvement of the vmPFC in controlling memory organization. We further demonstrate that the vmPFC exerts its control over HPC memory integration through direct projections to the medial entorhinal cortex (MEC), which can modulate MEC activity, directly change which HPC neurons are recruited to encode new information and alter the function of neurogliaform (NGF) inhibitory neurons in the dorsal CA1 (dCA1) to control memory integration. These vmPFC projections synapse onto MEC neurons in layers II, III and V that directly project to the dCA1. Together, these findings provide evidence for a biological mechanism through which prior knowledge can influence new memory formation and demonstrate how the vmPFC is directly involved in organizing memories in the HPC.

## Results

### vmPFC activity is modulated by the spatial and temporal contexts in which memories are acquired

**Fig. 1:**
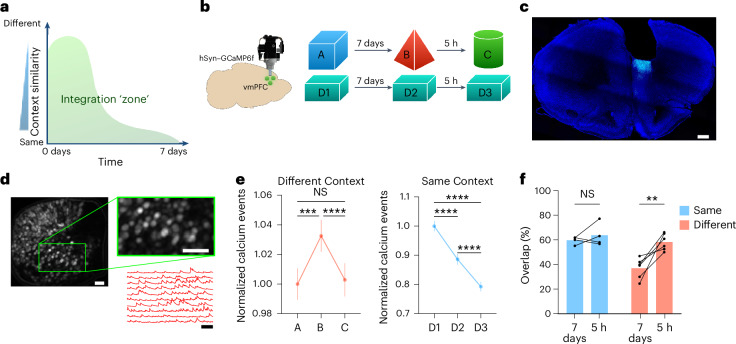
vmPFC activity is modulated by the time interval and context similarity between episodes. **a**, Graphical representation of two dimensions of memory organization and their influence on memory linking. **b**, Schematic representation of the experimental design. **c**, Representative confocal image showing GCaMP6f expression and GRIN lens placement in the vmPFC. Scale bar, 200 µm. **d**, Field of view and representative calcium trace from neurons recorded in the vmPFC. Scale bars, 50 µm and 1 min. **e**, Average of the number of calcium events per second of each cell across different episodes, normalized to the first episode. Statistical analysis was conducted using a Friedman test with two-tailed Dunn’s multiple-comparison test. NS, not significant. Different context group: *χ*^2^(2) = 29.33, *P* < 0.0001, *n* = 2,235, *N* = 6. Same context group: *χ*^2^(2) = 228.9, *P* < 0.0001, *n* = 1054, *N* = 4. **f**, Percentage of overlapping neurons in the vmPFC for different episodes. Statistical analysis was conducted using a multiple two-tailed paired *t*-test with Holm–Šídák correction. Different context: *t*(5) = 4.332, adjusted *P* = 0.0149, *N* = 6. Same context: *t*(3) = 0.8682, adjusted *P* = 0.4491, *N* = 4. In **e**,**f**, averages represent the mean ± s.e.m. *P < 0.05, **P < 0.01, ***P < 0.001 and ****P < 0.0001. Credit: Brain image adapted from Daniel Aharoni under a GNU GPL-3.0 license. Source data

Previous studies in humans and mice have demonstrated that the temporal and spatial contexts in which memories are encoded are critical dimensions for memory integration and organization^2,3,13–15^. In mice, memories involving the encoding of a spatial context are integrated when acquired 5 h but not 7 days apart, independently of the identity of the context^16–18^. In contrast, when these memories are encoded days apart, they are only integrated if the spatial context is very similar^16,19^, suggesting the existence of an ‘integration zone’ that is determined by the temporal and spatial contexts in which memories are acquired (Fig. 1a). Thus, to understand a possible role for the PFC in modulating memory organization according to these two dimensions, we recorded calcium transients in vmPFC neurons (here defined has the region of the PFC that contains the prelimbic (PL) and infralimbic (ILA) cortices^20^) using University of California, Los Angeles (UCLA) head-mounted fluorescence microscopes (that is, UCLA miniscopes) as mice visited the same or different contexts 5 h or 7 days apart (Fig. 1b–d).

**Extended data Fig. 1:**
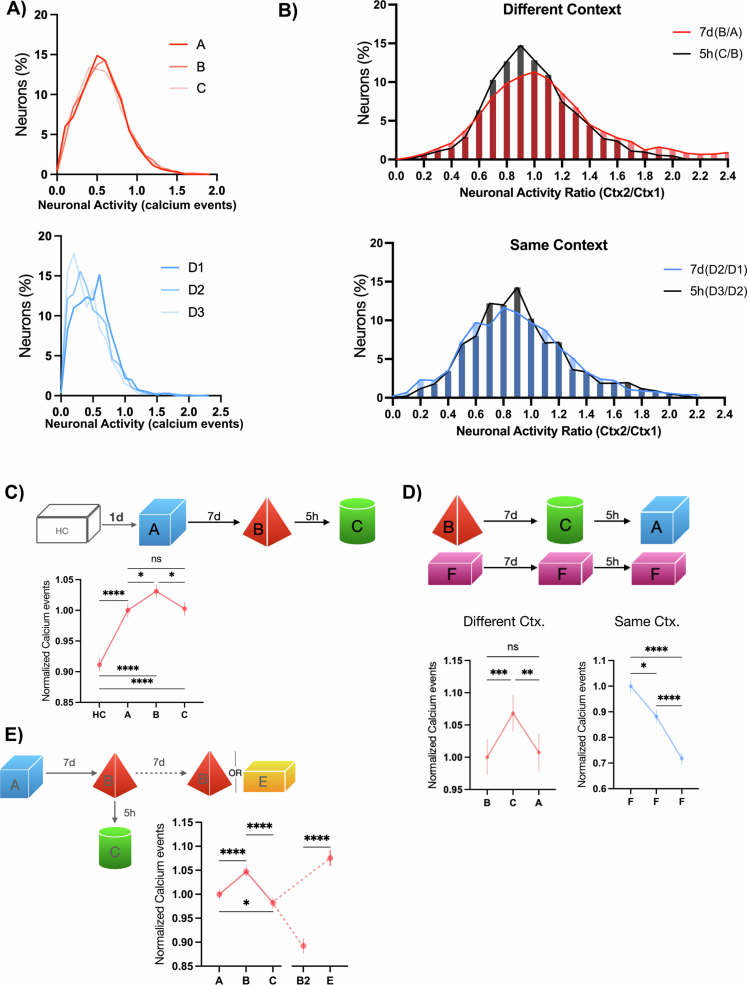
Analysis of vmPFC activity during contextual encoding. Related to Fig. 1. **A)** Histogram of neuronal activity during exposure to different contexts at different timepoints. Percentage of neurons VS Neuronal activity (calcium events). **B)** Histogram of ratio of activity between contexts. Percentage of neurons VS Activity ratio (calcium events) between sessions. Top panel: Activity ratio for different contexts B/A (7 days) and C/B (5 hours). Bottom panel: Activity ratio for same context D2/D1 (7 days) and D3/D2 (5 hours). For visualization purposes, outliers were removed using the ROUT method. **C)** Analysis of changes in activity from home cage to contextual encoding at different timepoints. Average of the number of calcium events per second of each cell across different episodes, normalized to context A. Friedman test, two-tailed Dunn’s multiple comparisons test χ2(3) = 188.5, p < 0.0001, n = 2267, N = 6. **D)** Changes in vmPFC activity are independent of context identity. Average of the number of calcium events per second of each cell across different episodes, normalized to the first episode. Friedman test, two-tailed Dunn’s multiple comparisons test. Different context group: χ2(2) = 15.71, p = 0.0004, n = 437, N = 3. Same context group: χ2(2) = 113.0, p < 0.0001 n = 509, N = 3 **E)** Sequential exposure to different or same context 7 days apart. Average of the number of calcium events per second of each cell across different episodes, normalized to the first episode. ABC: Friedman test, two-tailed Dunn’s multiple comparisons test, χ2(3) = 71.79, p < 0.0001, n = 2317, N = 6. B2-E: Mann-Whitney U = 539027, p < 0.0001, B2n = 1110, N = 3. En=1207, N = 3. Points represent mean ± SEM. *p < 0.05, **p < 0.01,***p < 0.001,****p < 0.0001. Source data

Analyses of the frequency of calcium events of individual cells (a proxy for neuronal activity) revealed striking differences in vmPFC activity across different conditions. When mice encoded different contexts, we observed a significant increase in the number of calcium events per second when the exposures were separated by 7 days but a significant decrease when the contexts were visited 5 h apart. In contrast, when the same context was visited, activity of vmPFC neurons decreased irrespectively of the time interval used (that is, 5 h or 7 days; Fig. 1e). We found different groups of neurons with different magnitudes of activity change, suggesting the existence of a heterogeneous population in the vmPFC during this task (Extended Data Fig. 1a,b). Importantly, this effect was independent of the identity and order of exploration of each context and persisted when mice sequentially visited three contexts 7 days apart (Extended Data Fig. 1c–e).

Similar to previous observations made in the HPC^16–18^, when mice explored two different contexts 5 h apart, the overlap between active neurons in the vmPFC was higher than when mice visited two different contexts 7 days apart. In contrast, when mice explored the same context, there was no difference in the percentage of overlapping active neurons with either 5-h or 7-day intervals (Fig. 1f). This result is different from what was previously reported in the HPC^16–18^ and suggests that the vmPFC maintains a more stable representation of spatial contexts that is less affected by representational drift.

Together, the results presented here demonstrate that vmPFC activity is modulated by both the time between spatial context exposures and the similarity between these contexts. In particular, the vmPFC appears to become highly engaged when two different contexts are explored 7 days apart (Fig. 1e), a condition that promotes maximal separation between memories^20^.

### vmPFC activity controls the integration of distant memories

**Fig. 2:**
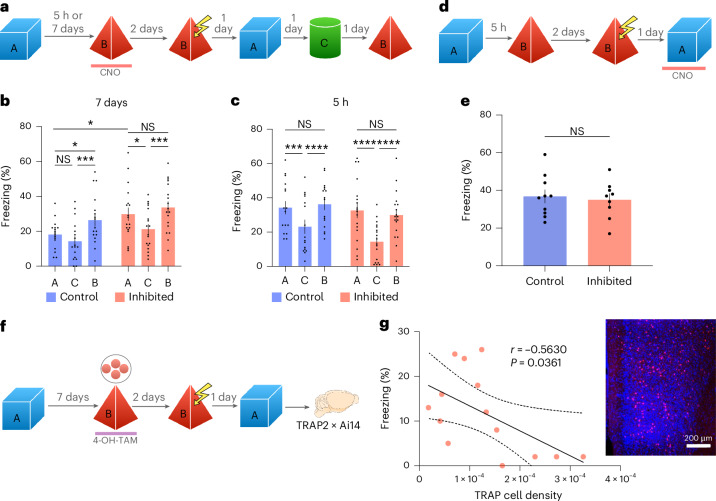
vmPFC activity controls memory integration at 7 days but not 5 h. **a**, Schematic representation of the behavioral protocol used to test the role of the vmPFC in memory linking. **b**,**c**, Freezing levels during exposure to context A, C or B after immediate shock in context B with (vmPFC inhibited) or without (control) vmPFC inhibition. Statistical analysis was conducted using a two-way RM ANOVA with two-tailed Tukey multiple-comparison test. For 7 days, main effect of context, *F*(2, 66) = 15.29, *P* < 0.0001; main effect of group, *F*(1, 33) = 6.208, *P* = 0.0179 (control, *N* = 17; inhibited, *N* = 18). For 5 h, main effect of context, *F*(2, 64) = 34.21, *P* < 0.0001 (control, *N* = 16; inhibited, *N* = 18). **d**, Schematic representation of the protocol used to test the role of vmPFC on the retrieval of linked memories. **e**, Freezing levels during reexposure to context A while vmPFC was inhibited in one group. Statistical analysis was conducted using a two-tailed unpaired *t*-test. *t*(17) = 0.3720, *P* = 0.7145 (control, *N* = 10; inhibited, *N* = 9). **f**, Schematic representation of the behavioral protocol used to evaluate the relationship between vmPFC activity and memory linking at 7 days in TRAP2×Ai14 mice. **g**, Linear regression between freezing levels in context A after the shock and number of TRAP cells per ROI area (pixels) in vmPFC. Pearson correlation: *r* = −0.5630, *P* = 0.0361 (*N* = 14). Inset: representative image of TRAP cells in the vmPFC. Scale bar, 200 µm. In **b**,**c**,**e**, bars represent the mean ± s.e.m. *P < 0.05, **P < 0.01, ***P < 0.001 and ****P < 0.0001. Source data

The results presented above suggest that the observed increase in vmPFC activity could be involved in separating unrelated memories encoded days apart. To test this hypothesis, we chemogenetically inhibited vmPFC neurons while mice performed a ‘memory linking’ task, where different memories are integrated when encoded 5 h apart but separated when encoded 7 days apart^16^. Mice were exposed to context A and, 7 days later, were exposed to context B while the vmPFC was inhibited in one of the groups using a pan-neuronal viral construct. Then, 2 days following exploration of context B, all mice received an immediate shock in this context (Fig. 2a). In this behavioral paradigm, memory integration is tested by measuring the magnitude of the conditioned response (freezing) in the context (context A) where mice were never shocked. Thus, 1 day following the immediate shock, mice were exposed again to context A and their freezing levels were recorded. Remarkably, mice that had the vmPFC inhibited during exploration of context B froze significantly more than control mice in context A and to levels similar to those displayed in context B, indicating robust memory integration. Importantly, this increase in the percentage of freezing was not because of memory generalization, as these mice exhibited significantly less freezing when exposed to a novel context C (Fig. 2b). The same behavioral results were also obtained when preferentially inhibiting excitatory neurons in the vmPFC, suggesting that this cell type is critical for the observed effect (Supplementary Fig. 1). Of note, vmPFC inhibition did not affect single memory encoding, total exploration time during inhibition or spontaneous social preference on a three-chamber assay, a set of findings that demonstrates the behavioral specificity of the vmPFC chemogenetic inhibition used here (Supplementary Fig. 2).

We also performed the same chemogenetic inhibition of the vmPFC when mice explored two contexts encoded 5 h apart, a condition that promotes memory integration^20^ and a natural reduction in vmPFC activity (Fig. 1e). This time, freezing levels of the vmPFC-inhibited group during reexposure to context A did not differ from control mice, indicating that the vmPFC is not involved in memory integration within this timeframe. Inhibition of the vmPFC did not affect either context memory encoding or generalization (Fig. 2c). Moreover, inhibition of vmPFC during retrieval of a linked memory (that is, two contexts encoded 5 h apart) also did not affect freezing levels (Fig. 2d,e), indicating that this brain region is also not involved in the retrieval of integrated memories. Additional analyses comparing the groups at 5 h and 7 days (Fig. 2b,c) revealed no significant differences in any context, except for the well-documented difference between 5 h and 7 days^16–18^ in the control group (Supplementary Fig. 3).

Our results (Figs. 1e and 2b) suggest that a natural increase in vmPFC activity, when mice visit two different contexts 7 days apart, might prevent memory integration. To further investigate this possibility, we used a double-transgenic mouse line (TRAP2×Ai14) that allows for the permanent expression of the tdTomato protein in neurons that are active during encoding of a given memory (TRAP neurons)^21^ upon injection of 4-hydroxy-tamoxifen (4-OH-TAM; administered interperitoneally). These mice were exposed to context A and, 7 days later, they were exposed to context B and injected with 4-OH-TAM to label c-Fos-positive (tdTomato-positive) cells activated during this context exposure. Then, 2 days later, mice received an immediate shock in context B and, the following day, we recorded their freezing levels in context A, as a measure of memory integration. The mice were killed 5 days later to quantify the number of tdTomato-positive cells in the vmPFC using confocal microscopy (Fig. 2f). Consistent with our hypothesis, we observed a negative correlation between the number of tdTomato-positive cells in the vmPFC and freezing levels during reexposure to context A (Fig. 2g), which was not observed for freezing levels in context A or B before the shock (Supplementary Fig. 4).

Together, these results strongly suggest that higher activity in vmPFC at the time of encoding of the second context (context B), 7 days after exposure to the first context (context A), reduces the likelihood that mice integrate both events into a mnemonic structure that modulates behavior during memory retrieval.

### vmPFC activity modulates the integration of memory ensembles in dCA1

**Fig. 3:**
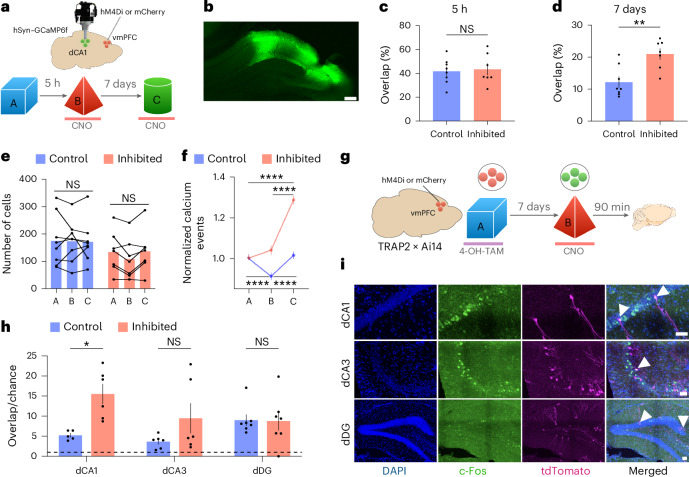
vmPFC activity controls ensemble overlap in the dCA1 during exposure to different contexts 7 days apart. **a**, Schematic representation of the protocol used to record calcium activity in the dHPC while inhibiting vmPFC activity. **b**, Representative confocal image of GCaMP6f expression in the dHPC. Scale bar, 200 µm. **c**,**d**, Percentage of overlapping active neurons in the dCA1 while mice explore different contexts separated by different time points with (inhibited) or without (control) chemogenetic inhibition of vmPFC. Statistical analysis was conducted using a multiple two-tailed unpaired *t*-test with Holm–Šídák correction. For 5 h, *t*(13) = 0.2783, adjusted *P* = 0.7851 (control, *N* = 8; inhibited, *N* = 7). For 7 days, *t*(13) = 3.626, adjusted *P* = 0.0061 (control, *N* = 8; inhibited, *N* = 7). **e**, Total number of cells per mouse for each group in the different contexts. Statistical analysis was conducted using a two-way RM ANOVA with two-tailed Šídák multiple-comparison test. *F*(2, 26) = 1.212, *P* = 0.3137 (*N* = 7–8 per group). **f**, Number of calcium events per second for each neuron across different contexts, normalized to the first episode. Statistical analysis was conducted using a Friedman test with two-tailed Dunn’s multiple-comparison test. Control: *χ*^2^(2) = 161.1, *P* < 0.0001, *n* = 2,177, *N* = 8. Inhibited: *χ*^2^(2) = 325.4, *P* < 0.0001, *n* = 1,583, *N* = 7. **g**, Schematic representation of the protocol used to identify neurons active during contexts A and B using TRAP2×Ai14 mice. **h**, Percentage of overlapping cells divided by chance levels for each subfield and each group. The dashed horizontal line indicates the change level. Statistical analysis was conducted using a multiple two-tailed unpaired *t*-test with Holm–Šídák correction. dCA1: *t*(9) = 3.601, adjusted *P* = 0.0171 (control, *N* = 5 for dCA1, 6 for dCA3 and 7 for dDG; inhibited, *N* = 6 for dCA1, 6 for dCA3 and 6 for dDG). **i**, Maximum-intensity projection of representative confocal images of the dHPC displaying neurons active in context A (red) and context B (green). Arrowheads indicate overlapping cells. Scale bars, 50 µm. In **c**–**e**,**h**, averages represent the mean ± s.e.m. *P < 0.05, **P < 0.01, ***P < 0.001 and ****P < 0.0001. Credit: Brain images adapted from Daniel Aharoni under a GNU GPL-3.0 license. Source data

**Extended data Fig. 2:**
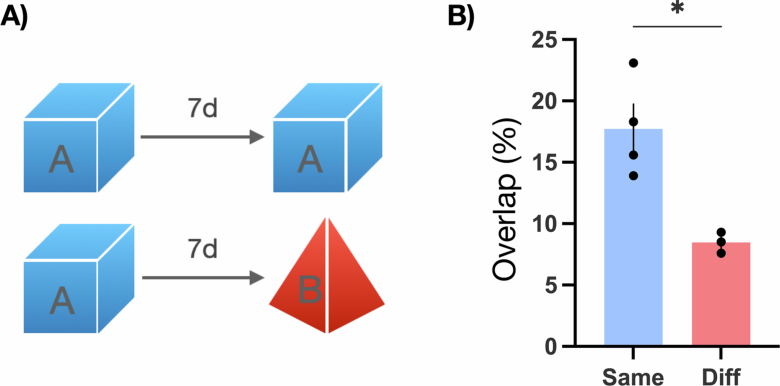
Representations of context in the HPC are more similar when mice visit the same context twice. **A)** Schematic representation of the protocol used to test the impact of context similarity on dCA1 representation. **B)** Percentage of overlapping neurons in the different groups. Two-tailed unpaired t-test t(5) = 3.840, p = 0.0121, Same N = 4, Diff N = 3. Bars represent mean ± SEM. *p < 0.05. Source data

**Extended data Fig. 3:**
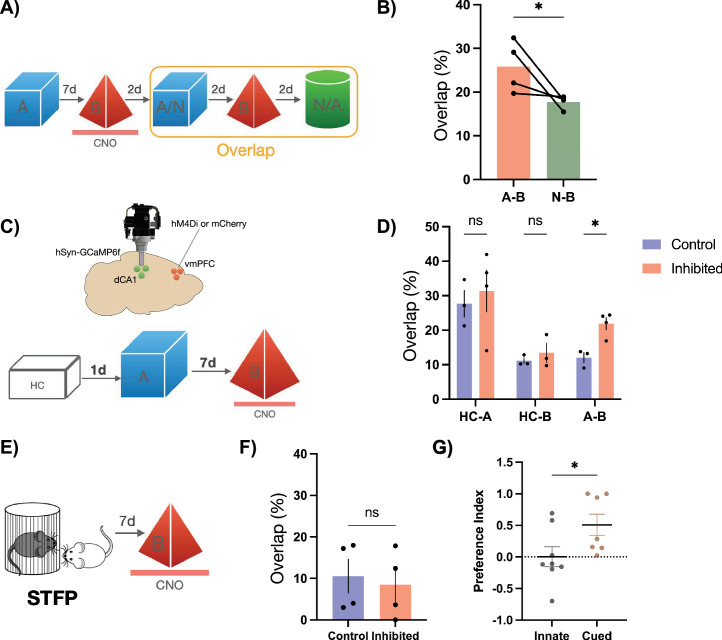
Analysis of the consequences of inhibiting vmPFC on HPC overlapping populations. Related to Fig. 3. **A)** Schematic representation of the protocol used to test wether memories linked due to vmPFC inhibition have more overlap during memory retrieval. **B)** Percentage of overlapping active neurons in the dCA1 during retrieval, following vmPFC inhibition during encoding of context B. One-tailed paired t-test. t(3) = 2.366, p = 0.0494, N = 4. **C)** Schematic representation of the protocol used to test if vmPFC inhibition links unrelated events. **D)** Percentage of overlapping active neurons in the dCA1between context A-B, Context A and home cage (HC-A), and Context B and home cage (HC-B). Multiple two-tailed unpaired t-test with Holm-Šídák correction. t(4) = 4.116, adjusted p = 0.0273, Control N = 3, Inhibited N = 4. **E)** Schematic representation of the protocol used to test if vmPFC inhibition links unrelated memories. **F)** Percentage of overlapping active neurons in the dCA1between the first memory (Social Transmission of Food Preference; STFP), and a new context. Two-tailed paired t-test. t(6) = 0.3607 p = 07307, N = 4 per group. **G)** Preference index between Cinnamon and Cocoa flavored food in naive mice (Innate) or mice that interacted with another mouse fed cinnamon-flavored food (Cued). Index = (Cin-Coc)/(Cin+Coc). Two-tailed paired t-test. t(13) = 2.175 p = 0.0487, Innate N = 8, Cued N = 7. In all panels, averages represent mean ± SEM. *p < 0.05. Credit: Brain images adapted from Daniel Aharoni under a GNU GPL-3.0 license. Source data

To understand which mechanisms allow for memory integration in the absence of vmPFC activity, we measured the percentage of overlapping neurons in the dCA1 when mice visited different contexts with or without vmPFC inhibition, as this overlap has been shown to mediate memory linking^16–18^. To this end, we chemogenetically inhibited the vmPFC while recording calcium activity in dCA1 neurons. Mice were exposed to different contexts, 5 h or 7 days apart, and vmPFC activity was inhibited during exposure to the second context in one group of mice (Fig. 3a,b). With a 5-h interval, inhibition of vmPFC activity did not affect the percentage of active overlapping neurons in the dCA1 (Fig. 3c). In contrast, with a 7-day interval, vmPFC inhibition triggered a significant increase in the percentage of overlapping dCA1 neurons between the two contexts (Fig. 3d), a result consistent with our behavioral results described above. This increase in overlap persisted during memory retrieval (Extended Data Fig. 3a,b). Moreover, this effect was not because of an unusual number of active neurons in the dCA1, as the total number of these cells detected upon vmPFC inhibition did not change significantly (Fig. 3e). The magnitude of the increase in overlapping ensembles was consistent with the magnitude of the difference normally observed when mice explored the same or different contexts 7 days apart (Extended Data Fig. 2), suggesting that this difference might be functionally important. Further investigation of the activity levels of single neurons revealed a significant increase in the number of calcium events per second in dCA1 when the vmPFC was inhibited during exploration of the second context (Fig. 3f). Interestingly, in a different group of mice, we observed that vmPFC inhibition specifically increased the overlap between neurons active during the exploration of two contexts visited 7 days apart but not between a context and the home cage (Extended Data Fig. 3c,d) or between a context and a different HPC-dependent task (social transmission of food preference) encoded 7 days apart (Extended Data Fig. 3e–g).

**Extended data Fig. 4:**
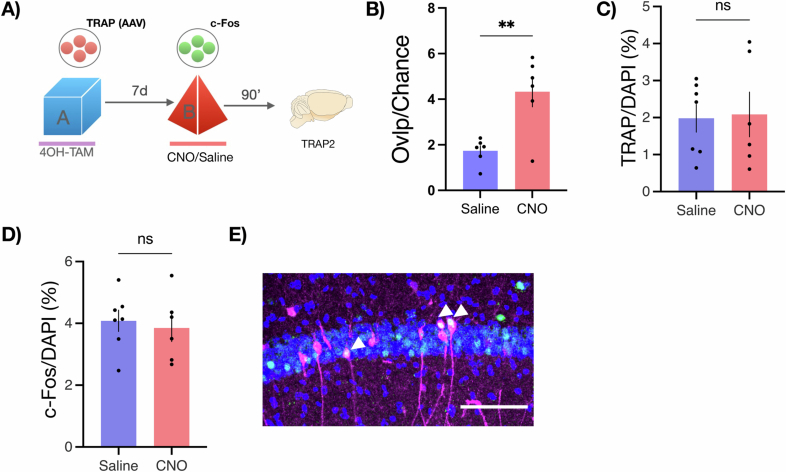
Quantification of c-Fos- and TRAP-positive cells in dorsal CA1 using TRAP2 mice and an AAV expressing hSyn-DIO-mCherry. **A)** Schematic representation of the protocol used to label neurons active during context A and context B in the dorsal CA1. **B)** Percentage of overlapping cells divided by chance levels for control (Saline) and inhibited (CNO) groups. Two-tailed unpaired t-test. t(10) = 3.685, p = 0.0042, N = 6 per group. **C)** Number of TRAP-positive cells (Red cells from context A) divided by the number of DAPI-positive cells in dCA1. Two-tailed unpaired t-test t(11) = 0.1522, p = 0.8818, Saline N = 7, CNO N = 6. **D)** Number of c-Fos-positive cells (Green cells from context B) divided by the number of DAPI-positive cells in dCA1. Two-tailed unpaired t-test. t(11) = 0.4204, p = 0.6823, Saline N = 7, CNO N = 6. **E)** Maximum-intensity projection of representative confocal images of the dorsal HPC displaying neurons active in context A (magenta) and context B (green). Arrows indicate overlapping cells. Scale bar: 100 µm Bars represent mean ± SEM. **p < 0.01. Source data

We next determined whether the neurons that participated in the increase in overlapping representations described above were part of the neurons encoding each memory, so-called engram cells^22^. We again used the TRAP2×Ai14 mice introduced above to label (with tdTomato) c-Fos-positive cells activated during the first episode (engram of context A) and performed immunohistochemistry to detect c-Fos-positive cells activated by the second context (engram of context B). Both contexts were explored 7 days apart and the vmPFC was inactivated in one group of mice during exploration of the second context (Fig. 3g). We measured these different populations of cells in dCA1, dCA3 and dorsal dentate gyrus (dDG) (Fig. 3h,i). Consistent with our results presented above, we observed a significant increase in the percentage of overlapping cells relative to chance levels in dCA1 when the vmPFC was inhibited. In contrast, there was no difference between groups in the other hippocampal subfields (Fig. 3h). Of note, there was also no difference in the number of tdTomato-positive or c-Fos-positive cells between groups in any of the analyzed subfields (Supplementary Fig. 5). Importantly, we used a third approach combining TRAP2 mice with viral expression of mCherry in the dCA1 and confirmed the findings just described (Extended Data Fig. 4). Together, the results presented here demonstrate that vmPFC activity specifically regulates memory integration in dCA1 and is crucial to control the integration of two related memories (that is, two spatial contexts) but is not necessary when two memories do not have features in common.

### Whole-brain survey of c-Fos expression reveals candidate brain regions involved in top-down control of memory integration in the HPC

**Fig. 4:**
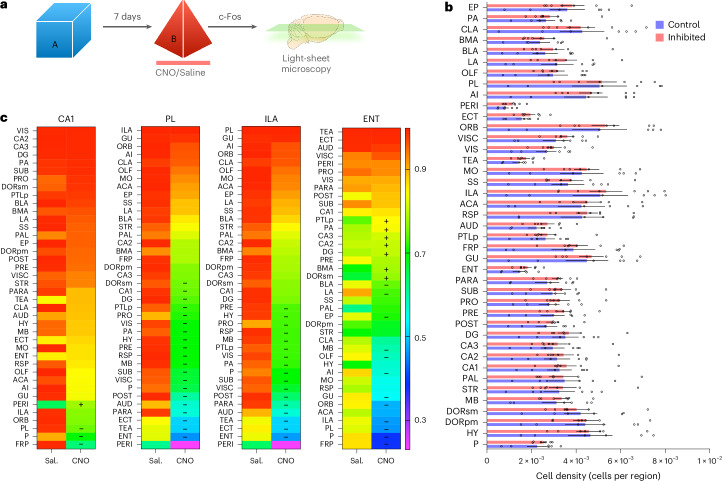
Whole-brain analysis of c-Fos expression reveals candidate regions for top-down control of HPC activity during memory integration. **a**, Schematic representation of the protocol used to quantify c-Fos-positive cells across the brain in mice with (CNO) or without (saline) inhibition of the vmPFC during exploration of context B. **b**, Average number of c-Fos-positive cells per region volume (in pixels). Statistical analysis was conducted using a multiple two-tailed unpaired *t*-test with Holm–Šídák correction. Saline, *N* = 6; CNO, *N* = 7. Bars represent the mean ± s.e.m. **c**, Pearson correlation coefficients sorted from highest to lowest in the inhibited group for HPC CA1, PL, ILA and entorhinal cortex (ENT). Saline, *N* = 6; CNO, *N* = 7. Overlaid ‘+’ or ‘−’ indicates a region that gained or lost a significant correlation upon vmPFC inhibition, respectively. Source data

While multiple studies suggest that PFC activity regulates memory processes in the HPC, the actual anatomical pathway through which this occurs is still largely unknown. To investigate how the inhibition of vmPFC described here could control memory integration in the HPC, we performed a whole-brain survey of neuronal activity (through quantification of c-Fos expression) after mice explored context B, 7 days after context A, with or without vmPFC chemogenetic inhibition (Fig. 4a).

Although we did not detect significant changes in any particular brain area after correcting for multiple comparisons (Fig. 4b), we observed altered correlations of c-Fos expression between several regions of interest (ROIs). When the vmPFC was inhibited, we detected a loss of significant correlation between the PL and ILA area in the vmPFC and many other brain regions, with particularly pronounced changes in areas of the parahippocampal region, including the perirhinal cortex, entorhinal cortex and ectorhinal cortex. Additionally, the hippocampal CA1 subfield showed a marked decrease in correlation with areas of the frontal cortex including the frontal pole, PL, ILA and orbital frontal cortex (Fig. 4c).

These observations led us to hypothesize that the vmPFC might be exerting its effects on the HPC through areas of the parahippocampal region. In particular, the entorhinal cortex is ideally suited for this function given its well-characterized bidirectional connectivity with the HPC and PFC and its proposed role in gating memory retrieval processes in the HPC^23^. Interestingly, the main changes observed in the entorhinal cortex included a decrease in correlation with the frontal pole, PL, ILA and orbital frontal cortex and an increase in correlation with areas of the hippocampal region (Fig. 4c). Further analyses of the separate regions of the entorhinal cortex revealed differences between the medial (MEC) and lateral (LEC) portions of this brain region. The MEC was more correlated with frontal areas in the control group, including the PL and ILA, and these correlations were lost upon vmPFC inhibition. In contrast, the LEC increased its correlation with all areas of the hippocampal region and other brain regions upon inhibition of the vmPFC (Supplementary Fig. 6).

### vmPFC neurons projecting to the MEC control the linking and integration of distant memories

**Fig. 5:**
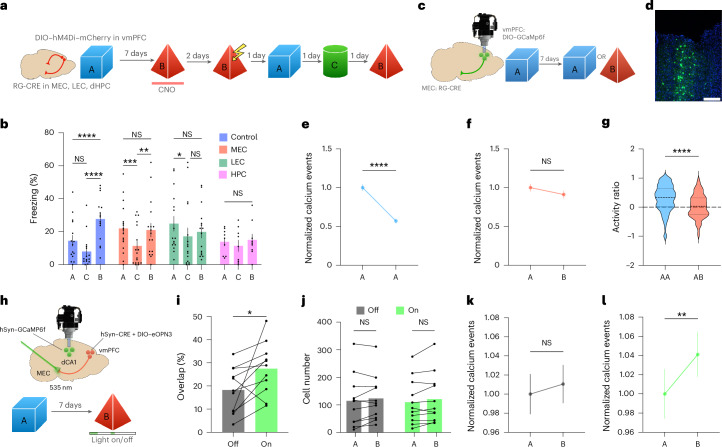
vmPFC–MEC projections control memory integration and ensemble overlap in the dHPC. **a**, Schematic representation of the protocol used to inhibit long-range projection neurons in the vmPFC. **b**, Freezing levels of different groups during exposure to contexts A, C or B following immediate shock in context B **b**. Statistical analysis was conducted using a two-way RM ANOVA with two-tailed Tukey post hoc test. Group × context interaction, *F*(6, 106) = 4.739, *P* = 0.0003 (control, *N* = 14; MEC, *N* = 16; LEC, *N* = 16; HPC, *N* = 9). **c**, Schematic representation of the protocol used to record calcium activity of vmPFC–MEC-projecting neurons. **d**, Representative confocal image showing expression of DIO–GCaMP6f and GRIN lens placement in the vmPFC. Scale bar, 200 µm. **e**,**f**, Average number of calcium events for each neuron across the same (**e**) or different contexts (**f**) normalized to the first episode. Statistical analysis was conducted using a two-tailed Wilcoxon matched-pairs test (*W*). Group AA, *W* = −21,145, *P* < 0.0001, *n* = 260, *N* = 4. Group AB, *W* = −3,993, *P* = 0.1866, *n* = 301, *N* = 4. **g**, Ratio of the calcium events for each neuron (episode 1 − episode 2/episode 1 + episode 2) in the different groups from **e**,**f**. Statistical analysis was conducted using a two-tailed Mann–Whitney test. *U* = 24835, *P* < 0.0001 (AA, *n* = 258; AB, *n* = 301). **h**, Schematic representation of the protocol used to inhibit vmPFC–MEC axonal terminals while recording calcium activity in the dHPC. **i**, Percentage of overlapping cells in the dHPC with (on) or without (off) inhibition of vmPFC–MEC projections. Statistical analysis was conducted using a two-tailed paired *t*-test. *t*(10) = 2.508, *P* = 0.0310, *N* = 11. **j**, Total number of active cells across context with (on) or without (off) inhibition. Statistical analysis was conducted using a multiple two-tailed paired *t*-test with Holm–Šídák correction. Off, *t*(10) = 1.479, adjusted *P* = 0.1700; on, *t*(10) = 2.046, adjusted *P* = 0.1312 (*N* = 11). **k**,**l**, Number of calcium events per second for each neuron across different contexts without (**k**) or with (**l**) vmPFC–MEC projection inhibition, normalized to the first episode. Statistical analysis was conducted using a two-tailed Wilcoxon matched-pairs test. In **k**, *W* = 7,196, *P* = 0.6637, *n* = 936, *N* = 11. In **l**, *W* = 82,980, *P* < 0.0001, *n* = 900, *N* = 11. In **b**–**g**,**i**–**l**, averages represent the mean ± s.e.m. *P < 0.05, **P < 0.01, ***P < 0.001 and ****P < 0.0001. Credit: Brain images adapted from Daniel Aharoni under a GNU GPL-3.0 license. Source data

**Extended data Fig. 5:**
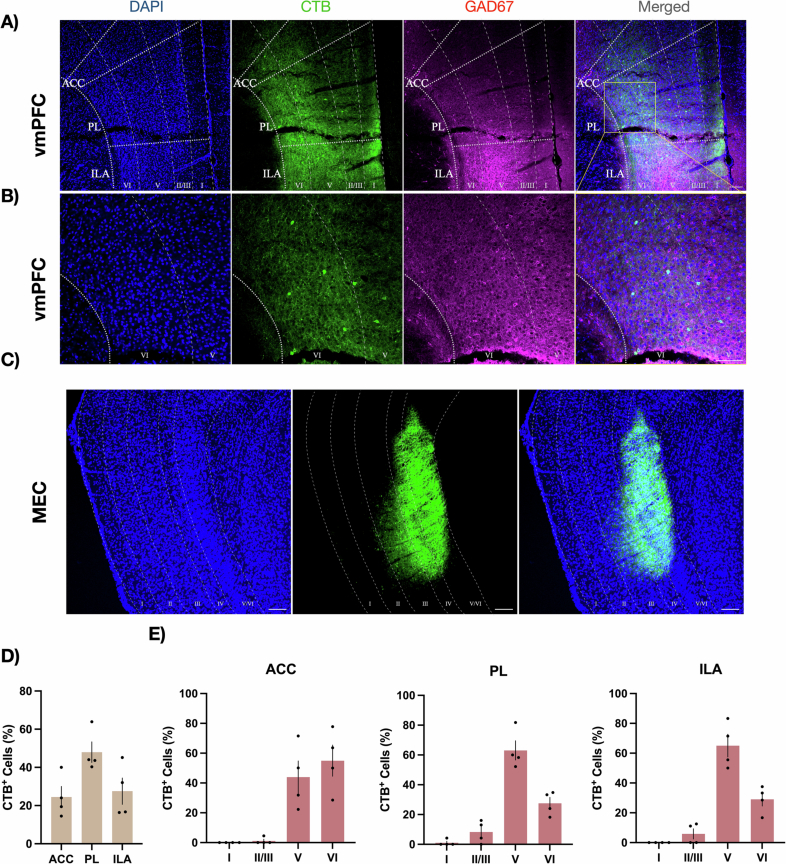
Characterization of vmPFC-MEC projecting neurons. **A)** Confocal image of vmPFC showing vmPFC-MEC projecting neurons identified by injecting CTB in the MEC (Green), and the inhibitory neuronal marker GAD67 (Magenta). **B)** Magnification of a particular area from the vmPFC image presented in B). **C)** Confocal image showing the site of CTB injection in the MEC. **D)** Quantification of the percentage of CTB-positive cells in different areas of the PFC, N = 4. **E)** Quantification of the percentage of CTB-positive cells per layer in different areas of the PFC, N = 4. Scale bar: 100 µm. No statistical analysis was performed. Bars represent mean ± SEM. Source data

Given the results presented above, we tested whether direct connections between the vmPFC and the entorhinal cortex or dorsal HPC (dHPC)^24^ could be controlling memory integration tested with a 7-day interval. To this end, we used an intersectional viral approach to chemogenetically inhibit vmPFC neurons that project to either the MEC, LEC or dHPC in different groups of mice. Mice were exposed to context A and, 7 days later, they were exposed to context B while specific vmPFC-projecting neurons were inhibited. Then, all mice underwent the same immediate shock in context B and retrieval protocol described above (Fig. 5a). Our results showed that inhibiting the vmPFC–MEC-projecting neurons was sufficient to recapitulate the memory linking results obtained after inhibition of the vmPFC. In contrast, inhibiting vmPFC–dHPC-projecting neurons appeared to directly affect the encoding of context B and inhibiting the vmPFC–LEC-projecting neurons increased freezing in both context A and in a novel context C, a sign of memory generalization (Fig. 5b). Identification of vmPFC neurons that project to the MEC using the viral strategy described above or a retrograde tracer (cholera toxin B) revealed that these neurons are more abundant in deeper layers of the vmPFC and do not express the glutamic acid decarboxylase 67 (GAD67) enzyme, an enzyme commonly used to identify inhibitory neurons. Moreover, we observed that the majority of projections from the vmPFC terminate primarily in layers II and V of the MEC (Extended Data Fig. 5 and Supplementary Fig. 7).

We then used a similar intersectional viral approach to record the activity of vmPFC–MEC-projecting neurons while mice explored either two different contexts (memory separation) or the same context (memory integration) 7 days apart (Fig. 5c,d). Similarly to the results presented above for general vmPFC activity, the vmPFC neurons projecting to the MEC showed a significant decrease in activity when mice explored the same context 7 days apart but not when they explored different contexts with the same time interval (Fig. 5e,f). Although we did not observe the same robust increase in activity detected in the entire vmPFC when mice visited two different contexts 7 days apart (Fig. 1e), there was a significant difference between the ratio of neuronal activity in both contexts across the different groups (Fig. 5g).

**Fig. 6:**
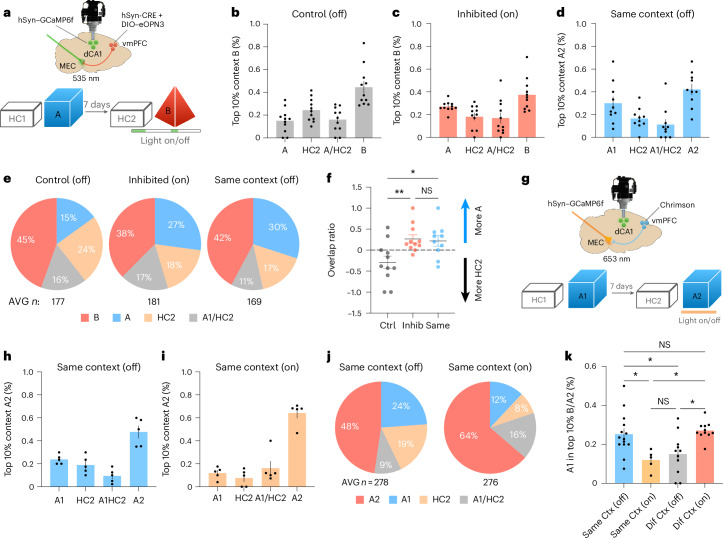
vmPFC–MEC projections control memory allocation and ensemble overlap in the dHPC. **a**, Schematic representation of the protocol used to inhibit vmPFC–MEC terminals and measure memory allocation and integration. **b**–**d**, Percentage of neurons in the top 10% most active cells in context B that are also in the top 10% active cells in context A, home cage before the second context (HC2), both context A and HC2 (A/HC2) and exclusively active in the second context (B or A2) for different groups (control, *N* = 11; inhibited, *N* = 11; same context, *N* = 10). **e**, Same as in **b**–**d**, but in a different representation. **f**, Percentage of highly active cells in context A and HC2 (top 10% most active) that are also on the top 10% most active cells in the second context (B or A2) (TopAB − TopHCB/TopAB + TopHCB). Statistical analysis was conducted using a one-way ANOVA with two-tailed Tukey multiple-comparison test. *F*(2, 29) = 6.181, *P* = 0.0058 (control, *N* = 11; inhibited, *N* = 11; same context, *N* = 10). **g**, Schematic representation of the protocol used to excite vmPFC–MEC terminals and measure memory allocation and integration. **h**,**i**, Percentage of neurons in the top 10% most active cells in context A2 that are also in the top 10% active cells in context A1 (A1), home cage before A2 (HC2), both context A1 and HC2 (A/HC2) and exclusively active in the second context (A2) for different groups (*n* = 5 per group). **j**, Same as **h**,**i**, but in a different representation. **k**, Direct comparison of the percentage of highly active cells form the first context (A1) that are also highly active in the second context (A2 or B top 10%) across all groups. Statistical analysis conducted using a one-way ANOVA with two-tailed Tukey multiple-comparison test. *F*(3, 37) = 6.089, *P* = 0.0018 (same context off, *N* = 14; same context on, *N* = 5; different context off, *N* = 11; different context on, *N* = 11). In **b**–**d**,**f**,**h**,**i**,**k**, averages represent the mean ± s.e.m. *P < 0.05 and **P < 0.01. Credit: Brain images adapted from Daniel Aharoni under a GNU GPL-3.0 license. Source data

**Extended data Fig. 6:**
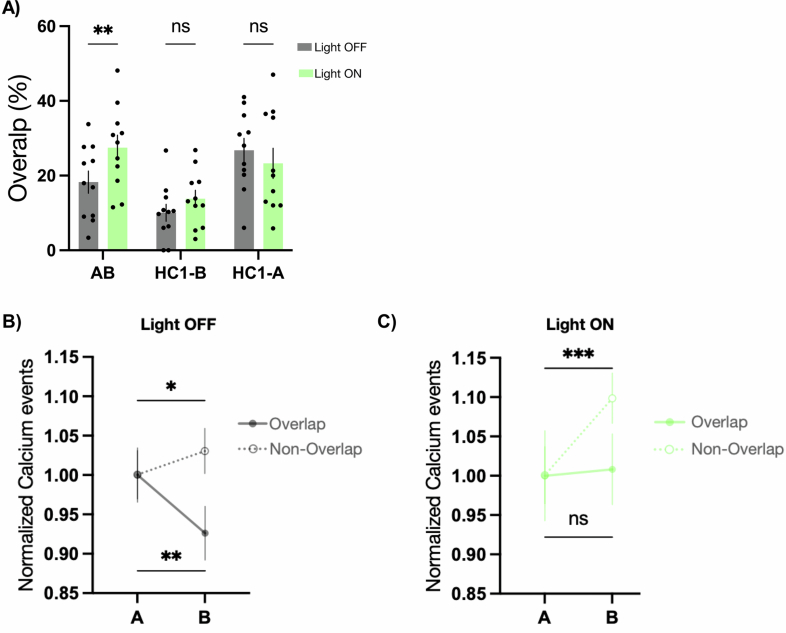
Detailed analysis of the overlapping population described in Fig. 5. Analysis of the activity of overlap and non-overlap neurons from Fig. 5k-l. **A)** The increase in overlapping neurons was specific to the visited contexts and not to a homage event recorded immediately before context A (Fig. 6 diagram). Two-way Repeated measures ANOVA, Two-tailed Šídák’s post-hoc test, Event x Light Interaction F (2, 20) = 6.961, p = 0.0051, N = 11. **B)** Number of calcium events per second for each neuron across different contexts without vmPFC-MEC projection inhibition normalized to the first episode. Two-tailed Wilcoxon matched-pairs test. Overlap: W = -1829, p = 0.0054, n = 109, N = 11. Non-Overlap: W = 6542, p = 0.0365, n = 308, N = 11. **C)** Number of calcium events per second for each neuron across different contexts with vmPFC-MEC projection inhibition normalized to the first episode. Two-tailed Wilcoxon matched-pairs test. Overlap: W = 155.0, p = 0.7974, n = 102, N = 11. Non-Overlap: W = 12537, p = 0.0003, n = 333, N = 11. In all panels, averages represent mean ± SEM. *p < 0.05, ***p < 0.001. Source data

Lastly, we asked whether activity of the vmPFC–MEC projections also controlled memory ensemble overlap in dCA1. Thus, we optogentically inhibited vmPFC–MEC terminals and recorded calcium activity in the dCA1 while mice explored different contexts 7 days apart. We used a mosquito-derived rhodopsin (eOPN3) that allows for long-lasting inhibition of axonal terminals following a brief light pulse^25^. To avoid spectral interference between the laser and the recording of calcium activity in the dCA1, axonal projections were inhibited for 2 min before mice were introduced in the context and again for 1 min at 5 min during context exploration (Fig. 5h). Calcium activity was also recorded for 5 min in the home cage before laser activation and context exploration (Fig. 6). We used a counterbalanced design where all mice visited two different pairs of contexts (each context of a pair separated by 7 days) with or without inhibition of vmPFC–MEC projections (light on and light off conditions, respectively; Methods). Remarkably, we observed a significant increase in the percentage of overlapping cells in the light on condition versus light off condition in almost all mice, indicating that inhibition of these axonal projections is sufficient to recreate the overall effect observed upon inhibition of vmPFC activity (Fig. 5I). Similar to the results reported in Fig. 3, we did not observe an overall increase in the number of active cells when light was delivered (Fig. 5j). Moreover, we did not observe an increase in the percentage of overlapping neurons between the second context and a home-cage event recorded immediately before the first context, despite high overlap between the latter two (Extended Data Fig. 6a). During exploration of the first pair of contexts, we observed a significant increase in the number of calcium events per second in individual dCA1 neurons when vmPFC–MEC projections were inhibited (Fig. 5k,l). Further analyses demonstrated that this effect was mainly driven by overlapping neurons that do not decrease their activity during the second context in the light on condition (Extended Data Fig. 6b,c).

Overall, the results presented here demonstrate that vmPFC neurons that project to the MEC are crucial to mediate behavioral and neuronal memory integration between events experienced several days apart.

### vmPFC–MEC projections modulate memory allocation in the HPC

Neurons that are highly excitable immediately before learning are preferentially recruited to encode new memories, a process known as memory allocation^26–30^. Our results demonstrate that, when two distant memories are integrated, neurons encoding the first memory are reactivated during encoding of the second, leading to the coallocation of the two memories into the same neurons (Fig. 3). These findings suggest that the vmPFC may control memory integration by modulating memory allocation mechanisms based on prior experiences.

To test this, we analyzed mice from Fig. 5h–l to determine whether inhibition of vmPFC–MEC projections altered recruitment of prelearning highly active neurons during encoding of context B (Fig. 6a). Recent studies suggest that, in the HPC, encoding of a spatial context relies on neurons that are highly active during context exploration^17,31^. Therefore, we examined whether the top 10% of neurons active in context B overlapped with neurons that were highly active in the home cage immediately beforehand (HC2) or during exploration of context A 7 days earlier (top 10%) (total number of neurons per mouse and condition in Supplementary Tables 1 and 2).

**Extended data Fig. 7:**
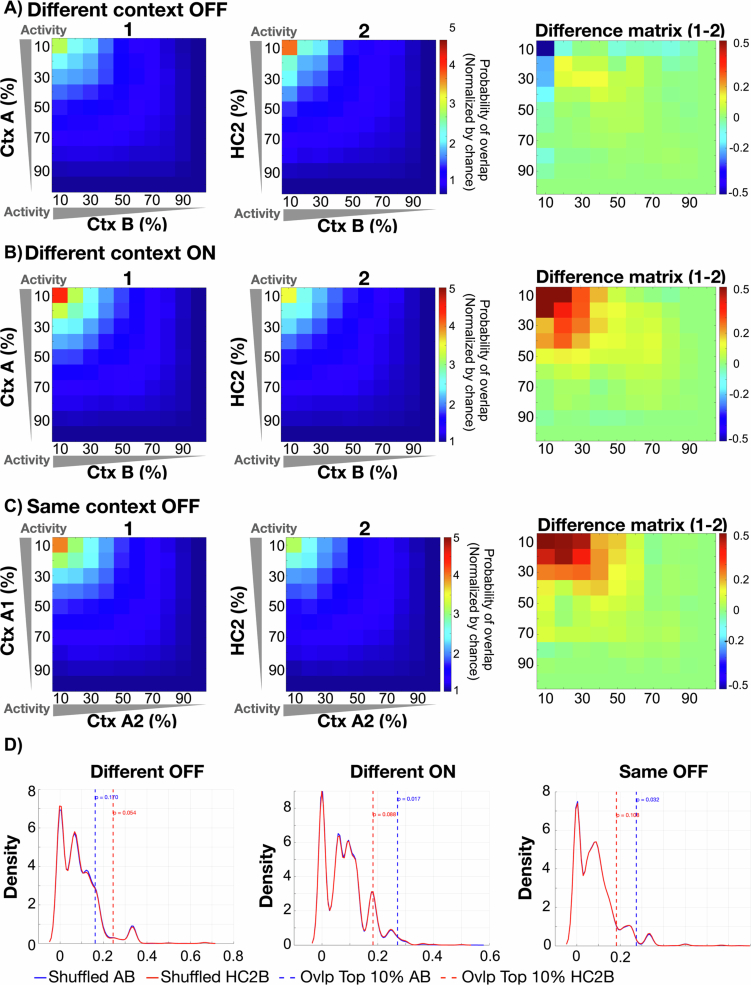
Analysis of overlap probability for different percentiles of activity. Related to Fig. 6. **A-C)** Matrices of overlap probability normalized to chance for different percentiles of activity in different groups. A difference matrix (matrix 1- matrix 2) is also shown, N = 11. **D)** Kernel density estimation (KDE) curves show the distribution of shuffled unique overlaps for top 10% most active neurons in Ctx A and Ctx B (blue), and HC2 and Ctx B (red), across all subjects. For each subject, unique overlap was defined as the fraction of neurons present in both sessions (CtxA-CtxB or HC2-CtxB), excluding neurons present in the 3 sessions (that is, CtxA∩HC2∩CtxB). Shuffled distributions were generated by randomly permuting CtxB activity 1,000 times. Dashed vertical lines indicate the group average of real (non-shuffled) unique overlaps for each comparison. p-values represent the proportion of shuffled overlaps that are greater than or equal to the observed real overlap (one-sided permutation test), indicating whether the real overlap exceeds chance. This analysis evaluates whether the overlap between top-firing neurons in early sessions and CtxB exceeds what is expected by chance, while excluding neurons active across the 3 sessions, N = 11. Source data

Under control conditions (light off), the top 10% of neurons active in context B consisted of ~15% context A neurons, ~24% home-cage neurons, ~16% neurons active in both and ~45% neurons unique to context B. Inhibiting vmPFC–MEC projections markedly shifted this distribution, increasing the contribution of context A neurons (27%) while reducing home-cage neurons (18%) and context B unique neurons (38%) (Fig. 6b–e). Interestingly, the distribution profile of active neurons observed in the inhibited group was very similar to the profile observed when these mice explored the same context 7 days apart (Fig. 6b–e; other percentiles of activity in Supplementary Fig 8; calculation of the probability of overlap for different combinations of percentiles of activity in Extended Data Fig. 7). Lastly, the ratio of home-cage to context A neurons differed significantly between all conditions (Fig. 6f).

**Extended data Fig. 8:**
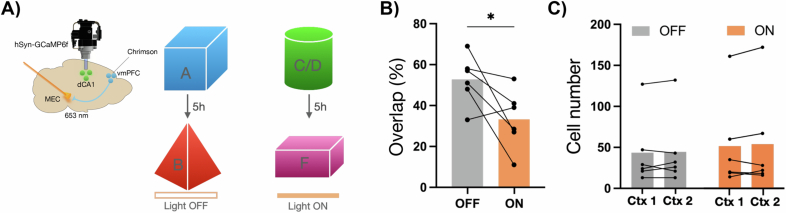
Activation of vmPFC-MEC projections decreases memory ensemble overlap when mice are exposed to two different context 5 hours apart. **A)** Schematic representation of the protocol used to activate vmPFC-MEC terminals and measure ensemble integration. **B)** Percentage of overlapping cells in the dCA1 with (ON) or without (OFF) activation of vmPFC-MEC projections. Two-tailed paired t-test, t(5) = 2.584,p = 0.0492, N = 6. **C)** Total number of active cells across context with (ON) or without (OFF) activation. Multiple paired t-test with two-tailed Holm-Šídák correction. OFF: t(10) = 0.04004, adjusted p = 0.9971, N = 6. ON: t(10) = 0.06898, adjusted p = 0.9971, N = 6. Credit: Brain images adapted from Daniel Aharoni under a GNU GPL-3.0 license. Source data

We next asked whether activity of vmPFC–MEC projections could also increase the separation between memories that would otherwise be integrated. To this end, we performed a similar experiment but activated vmPFC–MEC terminals using a ChrimsonR-td virus^32^ while mice explored the same context twice 7 days apart (Fig. 6g), a condition that normally promotes memory integration and a reduction in the activity of vmPFC–MEC projections (Fig. 5e). Again, we used a counterbalanced design where all mice visited two different pairs of contexts with or without activation of vmPFC–MEC projections during exploration of the second context (Fig. 6g). Remarkably we observed a significant reduction in the overlap of highly active neurons between the two sessions when these terminals were activated (from ~24% to ~12%) (Fig. 6h–j), the opposite of the effect observed when these projections were inhibited during exploration of different contexts (Fig. 6b–e). Similar to the result observed in the inhibition experiment, we also observed a reduction (from 19% to 8%) in the percentage of neurons that were highly active in the home cage before the second context exploration (HC2), suggesting that inhibition and activation of these projections do not exactly produce opposite effects on memory allocation. Interestingly, activating these projections also decreased ensemble overlap between different contexts explored 5 h apart (Extended Data Fig. 8), indicating that these projections can overcome local hippocampal integration mechanisms^16–18^.

Lastly, we compared the percentage of highly active neurons from the first context that were also highly active in the second context across all groups encoding contexts 7 days apart (Fig. 6k). Without optogenetic manipulation, overlap was higher when mice explored the same context (25%) than different contexts (15%). Activating vmPFC–MEC projections during repeated exposure to the same context reduced overlap to levels indistinguishable from those observed during exploration of different contexts (~15%), whereas inhibiting these projections during exploration of different contexts increased overlap to levels observed during repeated exposure (~25%).

Together, these results suggest that memory allocation is not solely dependent on neuronal intrinsic excitability or activity before a learning event^28,30^ but is influenced by prior experiences and can be bidirectionally modulated by vmPFC–MEC projections that determine whether memories are integrated or maintained as distinct representations.

### vmPFC–MEC projections modulate the activity of inhibitory cells in the stratum lacunosum moleculare through an MEC–HPC circuit

**Fig. 7:**
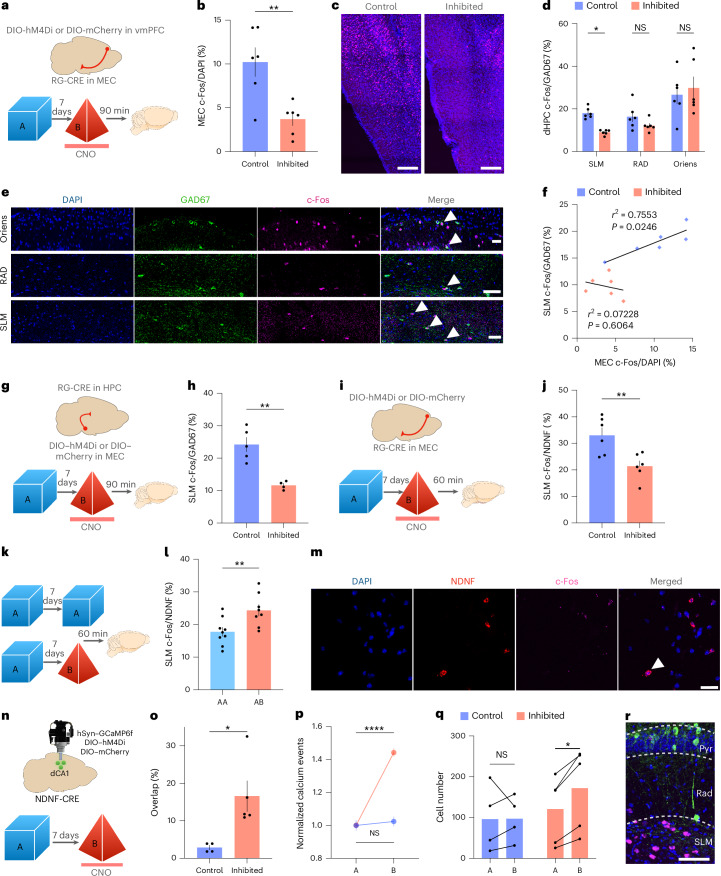
vmPFC–MEC projections control the activity of the MEC and of inhibitory cells in the SLM during memory integration. **a**, Schematic representation of the protocol used to investigate the impact of vmPFC–MEC projection inhibition on the activity of the MEC and inhibitory cells in the dHPC. **b**, Percentage of c-Fos-positive cells in the MEC of different groups. Statistical analysis was conducted using a two-tailed unpaired *t*-test. *t*(10) = 3.608, *P* = 0.0048, (control, *N* = 6; inhibited *N* = 6). **c**, Representative confocal images of c-Fos-positive cells in the MEC of different groups. Scale bars, 200 µm. **d**, Percentage of GAD67-positive cells that are c-Fos positive in different subfields of the dHPC. Statistical analysis was conducted using a multiple two-tailed unpaired *t*-test with Holm–Šídák correction. SLM, *t*(10) = 5.757, adjusted *P* = 0.000550; RAD, *t*(10) = 1.171, adjusted *P* = 0.465263; Oriens, *t*(10) = 1.136, adjusted *P* = 0.465263 (*N* = 6). **e**, Representative confocal images of DAPI-positive, GAD67-positive and c-Fos-positive cells in different subfields of the dCA1. Arrowheads indicate overlapping cells. Scale bars, 50 µm. **f**, Linear regression between the percentage of c-Fos-positive cells in MEC and GAD67/c-Fos double-positive cells in the SLM (*N* = 6 per group). **g**, Schematic representation of the protocol used to investigate the impact of MEC–HPC projection inhibition on the activity of SLM GAD67 neurons. **h**, Percentage of SLM GAD67-positive cells that are c-Fos positive in different groups. Statistical analysis was conducted using a two-tailed unpaired *t*-test. *t*(7) = 4.873, *P* = 0.0018 (control, *N* = 4; inhibited, *N* = 4). **i**, Schematic representation of the protocol used to investigate the impact of vmPFC–MEC projection inhibition on the activity of SLM NDNF-positive cells using RNAscope. **j**, RNAscope analysis measuring the percentage of NDNF-positive cells that are c-Fos positive in SLM. Statistical analysis was conducted using a two-tailed unpaired *t*-test. *t*(10) = 3.351, *P* = 0.0074 (control, *N* = 6; inhibited, *N* = 6). **k**, Schematic representation of the protocol used to investigate the activity of SLM NDNF cells upon exposure to the same or different context 7 days apart, using RNAscope. **l**, RNAscope analysis measuring the percentage of NDNF-positive cells that are c-Fos positive in SLM. Statistical analysis was conducted using a two-tailed unpaired *t*-test. *t*(15) = 2.995, *P* = 0.0091 (AA, *N* = 9; AB, *N* = 8). **m**, Maximum-intensity projection of RNAscope representative image showing DAPI-positive, NDNF-positive and c-Fos-positive cells. Arrowhead indicates overlapping cells. Scale bar, 50 µm. **n**, Schematic representation of the protocol used to investigate the impact of NDNF inhibition on memory integration. **o**, Percentage of overlapping cells in dCA1. Statistical analysis was conducted using a two-tailed unpaired *t*-test. *t*(7) = 2.937, *P* = 0.0218 (control, *N* = 4; inhibited, *N* = 5). **p**, Number of calcium events per second for each neuron across different contexts, normalized to the first episode. Statistical analysis was conducted using a two-tailed Wilcoxon matched-pairs test. Control: *W* = 2,136, *P* = 0.7986, *n* = 598, *N* = 4. Inhibited: *W* = 451,232, *P* < 0.0001, *n* = 1,236, *N* = 5. **q**, Total number of active cells across contexts. Statistical analysis was conducted using a multiple two-tailed paired *t*-test with Holm–Šídák correction. Control *t*(3) = 0.04161, adjusted *P* = 0.9694, *N* = 4. Inhibited, *t*(4) = 4.667, adjusted *P* = 0.0189, *N* = 5. **r**, Representative image of the HPC in NDNF-Cre mice. Blue, DAPI; green, GCaMP6f; red, mCherry. Scale bar, 100 µm. In **b**,**d**,**h**,**j**,**l**,**o**,**q**, bars represent the mean ± s.e.m. *P < 0.05, **P < 0.01 and ****P < 0.0001. RAD, stratum radiatum; Oriens, stratum oriens; Pyr, pyramidal layer. Credit: Brain images adapted from Daniel Aharoni under a GNU GPL-3.0 license. Source data

To further elucidate how the vmPFC controls ensemble overlap and memory allocation in the HPC, we used an intersectional viral approach to investigate how inhibition of vmPFC–MEC-projecting neurons impacted the activity in MEC neurons and local inhibitory circuits in the dHPC. Mice were exposed to context A and, 7 days later, exposed to context B, while vmPFC–MEC projecting neurons were chemogenetically inhibited in one group. We then performed immunohistochemistry to quantify c-Fos expression in the MEC and in different populations of dHPC inhibitory cells (Fig. 7a). We observed a striking reduction in the number of c-Fos-positive cells in the MEC when vmPFC–MEC-projecting neurons were inhibited during exploration of context B (Fig. 7b,c). Moreover, we observed a significant reduction in the number of double-positive GAD67/c-Fos cells in the stratum lacunosum moleculare (SLM) but not in the stratum oriens or stratum radiatum in the inhibited group (Fig. 7d,e). There was no difference in the total number of GAD67-positive or c-Fos-positive cells between groups (Supplementary Fig. 9a,b). To identify which inhibitory neuronal subtype(s) might be affected, we quantified c-Fos expression in somatostatin (SOM)-positive, parvalbumin (PV)-positive and vasoactive intestinal peptide (VIP)-positive inhibitory neurons across the dCA1. We did not detect significant differences between groups in any of these classes (Supplementary Fig. 9c). Interestingly, in control mice, c-Fos expression in the MEC positively correlated with the number of c-Fos/GAD67-positive cells in the SLM, suggesting that MEC activity may regulate local dCA1 disinhibition and memory integration (Fig. 7f).

**Extended data Fig. 9:**
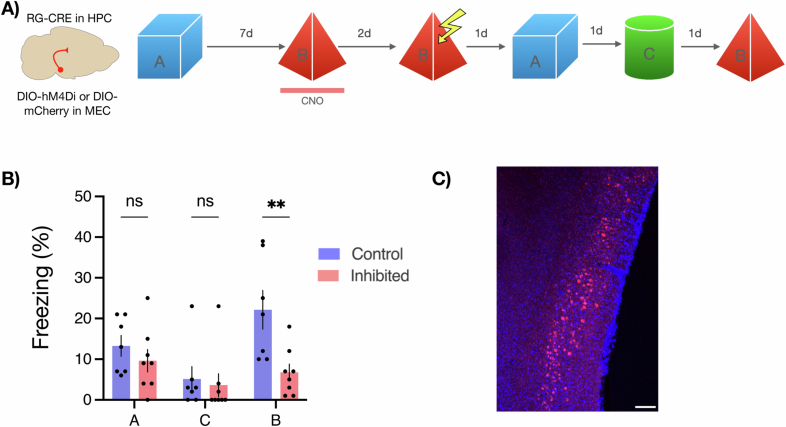
Inhibition of MEC-HPC projections impairs context encoding. **A)** Schematic representation of the protocol used to investigate the role of MEC-HPC projections in the linking of distal memories. **B)** Freezing levels in context A, C, and B, following immediate shock in context B. Two-way Repeated measures ANOVA, two=tailed Šídák’s post-hoc test, Context x Group Interaction F(2, 26) = 9.255, p = 0.0009, Control N = 7, Inhibited N = 8. **C)** Representative confocal image of mCherry expression in the MEC. Scale bar: 100 µm. Bars represent mean ± SEM. **p < 0.01. Source data

To test this possibility, we directly inhibited MEC–HPC-projecting neurons using a similar intersectional chemogenetic approach during exploration of context B, 7 days after context A, and quantified GAD67/c-Fos-positive cells in the SLM. We observed a significant decrease in double-positive cells in the inhibited group compared to controls (Fig. 7g,h). We next tested whether inhibition of these projections also influenced behavioral linking at 7 days. Using a behavioral paradigm similar to Fig. 5a, we chemogenetically inhibited MEC–HPC-projecting neurons during exploration of the second context. This manipulation impaired memory acquisition for context B and, therefore, precluded us from testing the impact of this manipulation on memory integration (Extended Data Fig. 9).

The observation that inhibitory neurons in the SLM are directly affected by inhibition of the vmPFC–MEC-projecting neurons suggests that this manipulation may affect local network computations and the flow of information to CA1 pyramidal cells. In particular, NGF inhibitory neurons in the SLM have been proposed to modulate the relative balance of encoding and retrieval processes by gating entorhinal cortex and CA3 inputs into CA1 (refs. ^33,34^), which could be relevant for the integration effect described here. Thus, we investigated whether NGF neurons were affected by vmPFC–MEC inhibition. To this end, we chemogentically inhibited vmPFC–MEC-projecting neurons and performed RNAscope to detect c-Fos and neuron-derived neurotrophic factor (NDNF) mRNA in the SLM, as NDNF has been identified as a marker of hippocampal NGF neurons^33–35^. Mice were exposed to context A and, 7 days later, to context B, while vmPFC–MEC-projecting neurons were inhibited in one group (Fig. 7i). We observed a significant reduction in NDNF/c-Fos double-positive cells in the inhibited group compared to controls (Fig. 7j).

We then asked whether the activity of NGF neurons was naturally modulated during memory integration. Mice were exposed to the same (integration) or different (separation) contexts 7 days apart and killed after the final exposure. RNAscope analysis revealed significantly fewer NDNF/c-Fos double-positive cells when mice explored the same context compared to different contexts (Fig. 7k–m), suggesting that these neurons are more active when memories need to be separated.

Lastly, to test a causal role for NGF neurons in memory integration, we chemogenetically inhibited NDNF-positive cells in the SLM of NDNF-Cre mice while recording calcium activity in dCA1 pyramidal neurons. As before, mice explored two different contexts 7 days apart and clozapine *N*-oxide (CNO) was administered during the second context (Fig. 7n). Inhibition of NDNF neurons increased ensemble overlap (Fig. 7o) and elevated calcium event rates (Fig. 7p), mirroring the results obtained following inhibition of vmPFC or vmPFC–MEC projections. Interestingly, this manipulation also slightly increased the total number of neurons recorded (Fig. 7q,r).

**Extended data Fig. 10:**
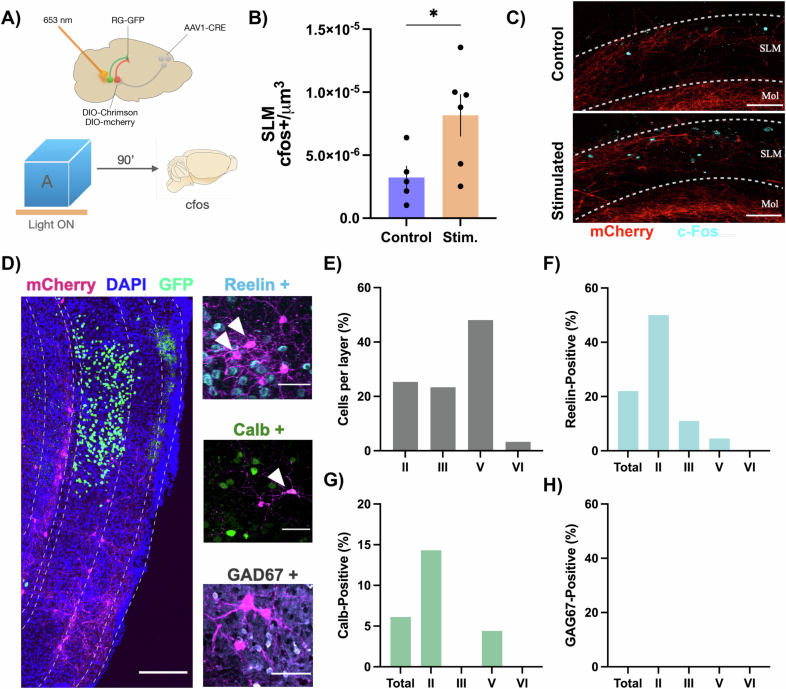
Characterization of the vmPFC-MEC-HPC circuit. **A)** Schematic representation of the protocol used. **B)** Number of c-Fos-positive cells in the SLM in the stimulated group (DIO-Chrimson) or control group (DIO-mCherry). Two tailed unpaired t-test t(9) = 2.465, p = 0.0359. Bars in represent mean ± SEM. *p < 0.05 **C)** Representative image of the HPC for each group. Red= mCherry, Cyan= c-Fos, SLM= Stratum Lacunosum Moleculare, Mol= Molecular layer of the DG. Scale bar: 100 µm. **D)** Representative z-stack image of the MEC. mCherry= MEC neurons that receive vmPFC inputs. GPF = MEC neurons that project to the dCA1. Reelin-, Calbindin, and GAD67-positive neurons in MEC and their overlap with neurons that receive vmPFC input. Scale bar large panel: 200 µm. Scale bar small panels: 50 µm **E)** Quantification of the number of neurons that receive vmPFC input on different layers of the MEC (n = 304 cells). **F-H)** Quantification of the percentage of vmPFC-MEC neurons that are positive for Reelin, Calbindin, and GAD67 across layers (total) or for each layer. Total number of mCherry cells counted: Reelin n = 50, Calbindin n = 98, Gad67 n = 137. Source data

To further define the vmPFC–MEC–HPC circuit involved in memory integration, we selectively targeted MEC neurons receiving vmPFC input by using an intersectional viral approach that included the injection of an anterograde transsynaptic virus in the vmPFC^36^ and expression of mCherry or an excitatory rhodopsin (ChrimsonR) in the MEC. Moreover, we injected a retrograde GFP virus in the dCA1 to identify MEC–dCA1-projecting neurons. Neurons in the MEC that receive direct input from the vmPFC were primarily detected in layers V (48%), II (25%) and III (23%). Approximately 6% of these neurons were Calbindin positive, 22% were Reelin positive and none were GAD67 positive. Notably, we observed mCherry-labeled projections in both the SLM and the molecular layer of the DG, suggesting that these neurons can directly direct influence dHPC processing (Extended Data Fig. 10). To test this hypothesis, mice were exposed to a novel context, while MEC neurons were optogenetically stimulated in one of the groups. Then, 90 min later, mice were killed and we quantified the number of c-Fos-positive neurons in the SLM, where the majority of NDNF cells are located. We observed a significant increase in the number of these neurons in the SLM in the stimulated group compared to the control group (Extended Data Fig. 10).

Together, these results indicate that NGF neuron activity is modulated by prior contextual experience, regulates ensemble overlap in dCA1 and is controlled by a vmPFC–MEC–HPC circuit, suggesting that they may be an integral part of memory organization processes controlled by the vmPFC.

## Discussion

Prior memories have long been known to influence novel encoding and change how memories are organized^1,4^. However, the biological mechanisms underlying these processes remain poorly understood. Here, we demonstrate that the vmPFC, a brain region involved in long-term memory storage^8^ and formation of memory schemas^37,38^, is recruited over time to modulate dCA1 ensemble overlap (through the MEC) and this determines how distant memories are integrated.

Hippocampal memories rely on prefrontal areas over time^8^ and interactions between these brain regions during learning are thought to be important for memory integration and organization^2–4,9–12^. Our results demonstrate that the vmPFC is involved in controlling the integration of context memories in the HPC through modulation of memory allocation mechanisms^26^. Activity of vmPFC neurons was highly modulated by the temporal distance and spatial context similarities between episodes, two factors that often determine memory integration. On average, the vmPFC appears to be more active during memory separation (7 days apart, different contexts) and less active during memory integration (5 h or 7 days, same context), suggesting a permissive role of this brain region during memory integration. Accordingly, chemogenetic inhibition of vmPFC activity, when mice encoded two different memories 7 days apart, led to the linking of both contexts and an increase in the number of overlapping neurons in the dCA1 encoding each context (observed with calcium imaging and c-Fos labeling). Interestingly, this increase in overlap was not observed between neurons active during contextual encoding and an event in the home cage or when mice encoded two different HPC-dependent tasks 7 days apart. These results suggest that the vmPFC is only necessary to control the integration of two memories when they share sufficient similarities.

Our results suggest that memories are preferentially integrated in the dCA1, while keeping some degree of segregation in the DG and CA3, perhaps to allow for the organization of memories according to their similarities while preserving the details of each episode, as previously proposed by computational models^39^. We should note that, when using TRAP2×Ai14 mice, we observed levels of overlap that were higher than expected by chance in the control group, which might have resulted from the low number of trapped cells in all groups. Indeed, when using a viral strategy to label neurons in TRAP2 mice, we observed overlapping levels that were consistent with the previous literature^40^ and still observed the same increase in overlapping neurons upon vmPFC inhibition, suggesting that the high levels observed in the double-transgenic mice did not affect the overall interpretation of the data. We further demonstrated that the vmPFC is naturally involved in controlling the integration of memories acquired 7 days apart but not memories acquired 5 h apart. This may indicate that a mature memory trace must develop in the PFC through systems consolidation^41,42^ for the PFC to selectively control memory integration in the HPC.

We uncovered the existence of a direct pathway from the vmPFC to the MEC that is able to selectively control memory organization in the HPC without affecting normal encoding processes. We should note that, because of the high variability of the behavioral data and lack of histological quantification of viral expression for every mouse in Fig. 5b, we cannot completely rule out the possibility of some contribution of vmPFC–LEC-projecting and vmPFC–HPC-projecting neurons in controlling memory integration at 7 days. However, we provide multiple lines of convergent evidence indicating that vmPFC–MEC projections are crucial to control memory integration. Specifically, we showed that activity of vmPFC–MEC projections is modulated by context familiarity at distant time points and inhibiting these projections (1) decreased activity (that is, c-Fos expression) in the MEC; (2) decreased the activity of putative NGF cells in the SLM; (3) increased ensemble overlap in the dCA1; and (4) led to memory integration at a time when this is not normally observed (that is, 7 days). In addition, activating vmPFC–MEC projections led to a decrease in ensemble overlap when memories are naturally integrated, suggesting that these projections can bidirectionally modulate memory integration in the HPC.

The vmPFC–MEC projections identified here appear to be mainly excitatory and target neurons in layers V, III and II of the MEC. In turn, these neurons directly project to the SLM (temporoammonic pathway) and DG molecular layer (trisynaptic pathway) of the HPC (Extended Data Fig. 10), pathways that are thought to be involved in memory encoding and retrieval, respectively^43^. Thus, the vmPFC is ideally positioned to control these processes through the MEC. In line with this possibility, the population of putative NGF cells studied here has also been shown to influence encoding and retrieval processes by modulating the activity of these pathways^33,44^. We demonstrated that these neurons can be directly modulated by vmPFC–MEC projections, MEC–HPC projections and are crucial to prevent inappropriate integration of different contexts encoded 7 days apart.

Lastly, our results suggest the existence of a previously unknown mechanism by which memory allocation is controlled. Previous studies indicated that memory allocation is dependent on the excitability or activity of neurons in a circuit, such that neurons with higher excitability or activity go on to encode a given memory^28,30^. However, examples when neuronal excitability or activity might not be sufficient to control memory allocation were shown previously^45,46^. Even though we did not directly measure intrinsic neuronal excitability in this manuscript, we demonstrated that vmPFC–MEC inhibition not only led to the reactivation of ensembles from prior memories but also resulted in the inhibition of neurons that were highly active before the learning event and otherwise would had been recruited to encode it^30^. Moreover, we observed that the percentage of highly active neurons that were naturally allocated to encode the second episode were dependent on whether mice had explored the same or a different context 7 days before. Together, these results reveal the existence of a novel memory allocation mechanism mediated by vmPFC–MEC–HPC interactions, which goes beyond the activity of individual neurons immediately before learning and is dependent on the content of prior memories.

Overall, the results reported here are consistent with the view that the PFC monitors and controls proper reactivation of memories in the HPC^47^. We provide evidence for a specific neuronal circuit that mediates this process and demonstrate that this circuit can control memory organization. These findings shed light on normal processes of memory organization and may also help to explain memory organization deficits associated with aging^48^ and several psychiatric disorders^5,6^ where PFC–HPC communication is known to be compromised^49–51^.

## Methods

### Subjects

Wild-type (WT) C57BL/6NTac (Taconic Farms) male and female 12–24-week-old mice were used in most experiments. Fos2A-iCreER (TRAP2) (RRID:IMSR_JAX:030323, Jackson Laboratory) mice were crossed with R26AI14/+ (AI14) (RRID:IMSR_JAX:007914, Jackson Laboratory) mice to generate double-transgenic TRAP2×Ai14 mice and maintained on a C57BL/6 Jackson background. Double-transgenic TRAP2×Ai14 or single-transgenic TRAP2 male and female 12–32-week-old mice were used in designated experiments. Male and female 12–32-week-old Ndnftm1.1(cre)Rudy/J (NDNF-Cre) (RRID:IMSR_JAX:030757, Jackson Laboratory) mice were used in designated experiments. All mice used in this study were group-housed with free access to food and water and maintained on a 12-h light–dark cycle. The temperature set point was 72 °F with humidity between 30% and 70%. Mice were single-housed for 1 week before behavioral experiments except following a surgical procedure, in which case they were single-housed for 2 weeks before the beginning of a behavioral experiment. Littermates were assigned to different experimental groups to prevent confounding effects of cage. All experiments were performed during the light phase of the cycle. All studies were approved by the Chancellor’s Animal Research Committee at UCLA.

### Memory linking task

All mice were handled and habituated to transportation and the behavioral room before initiating a behavioral experiment. Single-housed mice were first handled for three consecutive days in their colony room. Mice were handled by allowing them to freely rest or explore on the palm of the experimenter for 1 min on the first day, 1.5 min on the second day and 2 min on the third day. Then, mice were transported to the behavioral room every day for the next five consecutive days. Four cages (one mouse per cage) were transported at a time on a wheeled cart and covered with a black plastic bag to prevent mice from seeing their surroundings. Once inside the behavioral room, each mouse was handled for 2 min. All handling and habituation were performed at the same time of day (between 10 a.m. and 12 p.m.) and gloves were changed between each mouse.

Then, 1 day following the end of the habituation sessions, mice underwent a context linking experiment^16–18^. Mice were first exposed to context A for 10 min and, 5 h or 7 days later, were exposed to context B for 10 min, as detailed below. Then, 2 days later, mice were reexposed to context B, received a 2-s foot shock (0.75 mA) 10 s upon entering the context and remained in the context for an additional 30 s. During the next three consecutive days, mice were exposed to context A, a novel context C or context B and their freezing levels were recorded using an automated scoring system (Med Associates).

Each context consisted of a small chamber with particular visual, tactile and odor cues (Med Associates contextual fear condition system). Context 1 consisted of a smooth white plastic floor, a white plastic insert with round walls and a black insert ceiling and was cleaned with a 5% solution of Simple Green all-purpose cleaner. Context 2 consisted of a smooth white plastic floor topped with a roughed transparent floor, black triangle enclosure and one black–white checkered wall and was cleaned with a 5% solution of Windex glass cleaner. Context 3 consisted of horizontal metal rods (shocker) and a square enclosure with metal walls and was cleaned with 70% ethanol. All contexts were illuminated with white visible light. Contexts 1 and 2 were used for context A described in our experiments and were counterbalanced to be either the ‘linked context’ or a novel context. Context 3 was used as context B where mice received a mild foot shock.

### Miniscope recordings

All mice were handled and habituated to the behavioral room and the weight of the miniscope before starting a recording session. Then, 3 days after implantation of the baseplate, as described below, mice were handled in the colony room for 2 mins per day for five consecutive days. Then, 2 days later, mice were transported to the behavioral room as described above and handled for 2 min per day for five consecutive days. During the last 3 days of habituation, a V4 UCLA miniscope was attached to the baseplate and mice were allowed to freely move in their home cage for 2 min. On the last day of habituation, a 5-min recoding was performed in the home cage. Then, 2 days later, mice were allowed to explore context A for 10 min and, 7 days or 5 h later, explored context A, B or C while calcium activity was recorded in all sessions. In experiments where vmPFC was chemogenetically inhibited, as described below, CNO (5 mg kg^−1^) was injected 30 min before exposure to context B or C.

### Three-chamber social test

Mice with hSyn–hM4Di–mCherry, CamKII–hM4Di–mCherry or hSyn–Cherry in the vmPFC were tested on a classic three-chamber spontaneous social preference assay^52^. All mice were injected with CNO (5 mg kg^−1^) 30 min before being introduced into the three-chamber apparatus. Mice were first introduced in the central chamber and allowed to explore it for 5 min without access to the adjacent chambers. After this, mice gained access to the adjacent chambers and were allowed to explore the entire apparatus for 10 min. One of the outer chambers contained a conspecific inside a wire cup. The opposite outer chamber contained an object inside a wire cup. The object and conspecific chambers were counterbalanced across mice. Mouse behavior was recorded and the time spent on each chamber was manually scored during the 10-min exploration.

### Social transmission of food preference

Observer mice were tested for their relative preference for two new odor-laced foods after interacting with a demonstrator mouse. First, all mice were habituated to eating ground food in a bowl for 1 week in their home cage and were habituated to daily transport to the behavioral room. Demonstrator mice were then food deprived for 24 h and subsequently fed cinnamon-laced food (2% w/w) for 1 h. These mice were then introduced to the home cage of observer mice inside a wire cup and allowed to interact for 10 min. Observer mice were then food deprived for 24 h. The following day, observer mice were given a choice between cinnamon-laced food (2% w/w) or cocoa-laced food (1% w/w) in their home cage for 1 h. Total food consumption was determined by weighing each bowl before and after the trial. Innate preference for these two flavors was tested in naive mice.

### Chemogenetics

Mice were injected with CNO in saline (5 mg kg^−1^; Tocris, 4936) 30 min before being introduced to a context.

### Optogenetics

Following recovery from surgery, mice were handled and habituated as described above but an optic fiber cable was connected to the optic fiber during habituation. On the first day of recordings, calcium activity was first recorded for 5 min while mice were in their home cage. Immediately after, mice were exposed to a novel context (A) for 10 min and calcium activity was recorded. Then, 7 days later, calcium activity was again recorded in the home cage for 5 min followed by optogenetic stimulation of vmPFC–MEC terminals.

Optogenetic inhibition was achieved by delivering 535-nm laser light (500 ms, 0.5 Hz, 10–15 mA) for 2 min to half the mice while in their home cage. Immediately following light stimulation, mice were introduced to a novel context (B) and activity was recorded for 5 min. Recording was then stopped and laser light was again delivered for 1 min using the same pulse parameters. Activity was recorded for an additional 5 min before mice were returned to their home cage. Then, 10 days later, the procedure described above was repeated with new contexts (C and D) and light was delivered to the other half of mice.

Optogenetic activation was achieved by by delivering 653-nm laser light (10 ms, 20 Hz, 10–15 mA) for 10 min to half of the mice while they explored context A, 7 days after having visited the same context. Calcium activity in the dCA1 was recorded as described above. Then, 10 days later the procedure described above was repeated with a new context (B) and light was delivered to the other half of mice. This procedure was similar for the 5-h experiment but all mice visited two different pairs of contexts with light on or off.

### Stereotaxic surgery

Anesthesia was induced with 5% isoflurane in oxygen in an anesthesia chamber before mice were placed on a stereotaxic alignment system (Kopf Model 1900). Anesthesia was maintained using 1.5% isoflurane in oxygen. Petrolatum eye ointment was applied in both eyes (Puralube vet ointment) to prevent them from drying. Body temperature was maintained using a heating pad. Aseptic techniques were used throughout the entire surgery. Carprofen (5 mg kg^−1^) and dexamethasone (0.2 mg kg^−1^) were subcutaneously injected at the end of each surgery and for two (virus surgery) or seven (miniscope surgery) additional days. Following surgery, mice were allowed to recover over a heating pad in their home cage. Amoxicillin was provided in water for 2 weeks following the surgery.

For intracranial injection of viral vectors, a midline incision was made in the scalp and a small craniotomy was performed over the desired brain region using an air-driven dental drill (Henry Schein). A glass pipette was used in a nanoliter injector (World Precision Instruments) with a Micro4 Controller (World Precision Instruments) to inject viral vectors. All coordinates for injections were relative to Bregma unless otherwise specified. For vmPFC viral injections, +1.8 mm anterior–posterior (AP), ±0.35 mm medial–lateral (ML) and −2.1 mm dorsal–ventral (DV). For dHPC, −2.1 mm AP, ±2 mm ML and −1.65 DV. For MEC, −4.85 mm AP, ±3.4 mm ML and −3.5 mm DV. For LEC, −3.65 mm AP, ±4 mm ML and −4.25 mm DV. Viral vectors were injected at a rate of 50 nl min^−1^. After injection, the scalp was closed using 9-mm wound clips (Clay Adams) that were removed after 8 days.

For in vivo imaging of calcium activity, a GRIN lens was implanted over the target region 30 min following viral injection. To this end, the skull was scorched and a 1.2-mm-diameter screw was implanted over the posterior parietal cortex on the opposite side of the lens to improve adherence of dental cement. A circular craniotomy (diameter: 1 mm) was then carefully performed over the target region. Part of the neocortex was carefully aspirated using a blunt hypodermic needle (27 G) while artificial cerebrospinal fluid was applied to prevent drying. Once bleeding stopped, a 1-mm GRIN lens was slowly lowered to the target position. For vmPFC, the lens was implanted at +1.8 mm AP, −0.5 mm ML and −1.9 mm DV. For dHPC, the lens was implanted at −2.1 mm AP, −2 mm MLand −1.35 mm DV (from the top of the craniotomy). The GRIN lens was secured to the skull using cyanoacrylate glue and dental cement (Ortho-Jet, LANG). Kwik-Sil (World Precision Instruments) was used to cover the GRIN lens. Then, 2 weeks later, a baseplate was cemented over the GRIN lens using the V4 miniscope to determine the position that ensured the best focal plane.

For experiments involving in vivo calcium imaging and optogenetics, surgeries were performed in two phases. First, all viruses were injected as described above. Then, 1 week later, both the GRIN lens and the optic fiber were implanted on their respective target areas. The optic fiber was implanted over the MEC at an angle to ensure sufficient space between the miniscope and optic fiber (−4.85 mm AP ±3.4 mm ML and −3 mm DV; 16° angle on AP axis towards posterior).

### Viral vectors

The viral vectors described below were injected using the coordinates and methodology described above.

For vmPFC, the vectors consisted of 200 nl of pAAV-hSyn-mCherry (Addgene, 114472-AAV5), 200 nl of pAAV-hSyn-hM4D(Gi)-mCherry (Addgene, 50475-AAV5), 400 nl of pAAV.Syn.GCaMP6f.WPRE.SV40 (Addgene, 100837-AAV1), 400 nl of pAAV.CAG.Flex.GCaMP6f.WPRE.SV40 (Addgene, 100835-AAV1), 250 nl of pAAV-hSyn-DIO-hM4D(Gi)-mCherry (Addgene, 44362-AAV8), 250 nl of AAV8-hSyn-DIO-mCherry (Addgene, 50459-AAV8), 200 nl of pAAV-CaMKIIa-hM4D(Gi)-mCherry (Addgene, 50477-AAV5), 200 nl of pOTTC1484-pAAV SYN1 HA-hM4D(Gi) (Addgene, 121538-AAV5), 200 nl of pAAV-hSyn1-SIO-eOPN3-mScarlet-WPRE (Addgene, 125713-AAV5), 250 nl of pENN.AAV.hSyn.Cre.WPRE.hGH (Addgene, 105553-AAV1) and 250 nl of pAAV-Syn-ChrimsonR-tdT (Addgene, 59171-AAV5).

For dHPC, the vectors consisted of 400 nl of pAAV.Syn.GCaMP6f.WPRE.SV40 (Addgene, 100837-AAV1), 300 nl of pAAV-EF1a-Cre (Addgene, 55636-AAVrg) and 300 nl of AAV8-hSyn-DIO-mCherry (Addgene, 50459-AAV8).

For MEC, the vectors consisted of 350 nl of pAAV-EF1a-Cre (Addgene, 55636-AAVrg), 400 nl of pAAV-hSyn-DIO-hM4D(Gi)-mCherry (Addgene, 44362-AAV8), 400 nl of AAV8-hSyn-DIO-mCherry (Addgene, 50459-AAV8) and 400 nl of` pAAV-Syn-FLEX-rc[ChrimsonR-tdTomato] (Addgene, 62723-AAV5).

For LEC, the vector consisted of 350 nl of pAAV-EF1a-Cre (Addgene, 55636-AAVrg).

### Immunohistochemistry

First, 90 min after behavior, mice were transcardially perfused with PBS followed by 4% paraformaldehyde (PFA) in 0.1 M phosphate buffer. Brains were collected, incubated overnight in 4% PFA and then transferred to PBS for storage at 4 °C. Brains were sliced at 50 µm using a vibratome (Leica VT100S) or incubated in a 30% sucrose solution to be sliced at 50 µm using a cryostat (Leica CM1850).

Free-floating brain slices were incubated with a blocking solution containing 0.3% Triton X-100 in PBS and 10% normal goat serum (Vector Laboratories, S-1000) at room temperature for 1 h to minimize nonspecific antibody binding in the subsequent steps. Primary and secondary antibodies were diluted in the same blocking solution. Free-floating sections were incubated with the primary antibody overnight at 4 °C with constant shaking, repeatedly washed in PBS and then incubated with the secondary antibody for 2 h at room temperature with constant shaking. Slices where then incubated with DAPI (Invitrogen; 1:1,000) for 20 min and repeatedly washed in PBS before being mounted on glass slides (VWR Superfrost Plus) using DAPI Fluoromount-G mounting media (Southern Biotech).

Primary antibodies were as follows: rabbit anti-c-Fos (Cell Signaling, 9F6, 2250; 1:700), chicken anti-RFP (Synaptic Systems, 409006; 1:700), mouse anti-GAD67, clone 1G10.2. (EMD Millipore, MAB5406; 1:1,000), rabbit anti-cholera toxin B antibody (Abcam, ab34992; 1:1,000), mouse anti-PV (Sigma-Aldrich, P3088; 1:1,000), guinea pig anti-VIP (Synaptic Systems, 443005; 1:100), rat anti-SOM (EMD Millipore, MAB354; 1:100), guinea pig anti-Calbindin D28k (Synaptic Systems, 214004; 1:700) and goat anti-Reelin polyclonal antibody (Invitrogen, PA5-47537; 1:1,000).

Secondary antibodies were as follows: goat anti-rabbit Alexa Fluor 647 (Invitrogen, A-21244; 1:1,000), goat anti-rabbit Alexa Fluor 488 (Invitrogen, A-11008; 1:1,000), goat anti-chicken Alexa Fluor 594 (Invitrogen, A-11042; 1:1,000), goat anti-mouse Alexa Fluor 488 (Invitrogen, A-11001; 1:1,000), goat anti-guinea pig Alexa Fluor 568 (Invitrogen, A-11075; 1:1,000), goat anti-rat Alexa Fluor 594 (Invitrogen, A-11007; 1:1,000) and donkey anti-goat Alexa Fluor 647 (Invitrogen, A-21447; 1:1,000).

### RNAscope

Brains were collected 60 min after behavior and fast-frozen in optimal cutting temperature compound by dry ice without PFA fixation. Frozen brains were sliced at 15 µm using a Leica Cryostat. In situ hybridization was performed using the RNAscope fluorescent multiplex reagent (ACD, 323120) according to the instructions from the manufacturer. Probe-Mm-Ndnf (ACD, 447471) and Probe-Mm-Fos (316921) were used for mRNA labeling.

### Confocal microscopy

Images were acquired using a Nikon A1 laser scanning confocal microscope and analysis was performed using NIS-Elements AR Analysis software (Nikon; version 4.40.00).

Slices from TRAP2×Ai14 mice (Fig. 2e) were imaged at ×10 magnification and 6–8 slices per mouse were used to automatically determine the number of tdTomato-positive cells in the vmPFC.

Slices from TRAP2×Ai14 mice (Fig. 3h) were imaged using at ×20 magnification with a *z* step of 2 µm for a total of 27 steps per slice. A maximum *z* projection was generated for each field of view and a total of four slices per mouse were used to determine the number of DAPI-positive, c-Fos-positive and RFP-positive cells in the HPC. In this case, DAPI-positive cells were automatically counted but c-Fos-positive, RFP-positive and double-positive cells were obtained by averaging the manual counts of 2–3 experimenters blind to the experimental conditions. Chance levels were calculated as (c-Fos/DAPI) × (TRAP/DAPI).

Slices from WT mice (Fig. 7b) were imaged at ×20 magnification with a *z* step of 2 µm for a total of 27 steps per slice. A total of four slices per mouse was used to determine the number of c-Fos-positive, PV-positive, SOM-positive, VIP-positive and GAD67-positive cells in the dCA1. Counts were obtained by averaging the manual counts of 2–3 experimenters blind to the experimental conditions. DAPI was automatically counted. MEC DAPI and c-Fos were also automatically counted.

For RNAscope (Fig. 7f), brain slices were imaged at ×20 magnification and 2–3 slices per mouse were used to manually count NDNF-positive and C-Fos-positive cells in the SLM. Final counts were obtained by averaging the scores of three experimenters blind to the experimental conditions.

### Miniscope analysis

One-photon calcium imagining was performed using the UCLA V4 miniscope (https://github.com/Aharoni-Lab/Miniscope-v4)^17^. Specifically, during recordings, digital imaging data were sent from the complementary metal–oxide–semiconductor (CMOS) imaging sensor (Aptina, MT9V032) to custom data acquisition electronics and a USB host controller (Cypress, CYUSB3013) over a lightweight, highly flexible coaxial cable. Images were acquired at 30 frames per second, using a display resolution of 600 × 600 pixels (1 pixel = 1–2 µm) and saved into uncompressed mp4v files. The analysis pipeline was written in MATLAB R2020b using the ConcatMiniscope pipeline^53^. The algorithm first runs the NoRMCorre algorithm for motion correction (rigid registration) of individual sessions from a single animal^54^, followed by the alignment and normalization of brightness across sessions and concatenation into a single long video. Alignment was performed aligned using a semiautomatic alignment tool based on the ‘imregtform’ function (MATLAB R2020b, image-processing toolbox) followed by manual checking of landmarks (usually blood vessels). Individual neurons were identified and extracted using the CNMF-E algorithm^55^. During motion correction, videos were 2× spatially downsampled using the default built-in NoRMCorre protocol. During CNMF-E initialization, videos were further 2× spatially downsampled and 5× temporally downsampled. The quality of neuron extraction was then verified using a MATLAB R2020b custom-made neuron deletion GUI. We excluded the detected putative neurons exhibiting ROI morphology or calcium trace abnormalities or incoherencies between the calcium trace peaks and the expected correspondent fluorescence increases in the video.

For quantification of neuronal activity across sessions, after the detection of individual neurons, CNMF-E projects their raw activity as a relative brightness of each ROI and compiles them into the variable neuron.C_raw. To quantify the activity of the same neuron across multiple sessions, we first separated the neuron.C_raw from each session and deconvolved the calcium signal form each neuron using an empirical method based on multiple one-dimensional convolutions of the raw signal with gaussian kernels. According to the assumption that true calcium transients are represented by steep increases in fluorescence followed by slow exponential decay, we devised a fast method to separate steep increases in brightness that are related to actual calcium transients from the background noise using a nonparametric analysis. Briefly, we convolved the raw data separately with two Gaussian kernels with s.d. of 50 and 100 ms. Then, for each convolution, we performed a 3× decimation (each data point comprised ~100 ms) and calculated the first derivative of the data. The distribution of first derivatives represents the distribution of steepness of calcium increases within 100-ms time windows, well within the timeframe of the calcium indicator. For each convolution with each of the two kernels, we calculated a threshold as the third quartile plus 1.5× the interquartile range (~3 s.d. above the mean in a normal distribution). We only considered actual calcium transients the data points with steepness that were higher than both thresholds, which were deemed as 1, while the remaining data points were deemed as 0. As an additional quality-control step, putative noisy neurons were automatically detected and then manually removed. Neurons were deemed noisy and sorted out for further inspection if their peak-to-noise ratio was lower than 20 or if the distribution of the noise showed a difference between the first and third quartiles higher than 200%. The number of calcium events (putative firing rates) per second for each neuron was calculated as the number of 1 values per time interval and their average on each session was determined by the total number of 1 values divided by the number of data points. The top 10% of active cells (Fig. 6) were identified by sorting the average number of calcium events per second on a given session from highest to lowest.

For overlap quantification, for the determination of the percentage of overlapping ensembles between two sessions, the 10-min recordings of each session were individually analyzed, as described above (without concatenation). Recordings from both sessions in the same mouse were first aligned using the spatial footprints (neuron.A, output from CNMF-E) of each one of the detected cells for individual sessions. The centroid distance and spatial correlation were calculated for all cell pairs. Cell pairs from different sessions were considered to match if their spatial correlation was at least 0.8 and their centroid distance was 5 pixels or less. Overlapping percentages between two sessions were calculated as the number of matched cells over the average of the total number of detected cells in each one of the two sessions. Overlapping index = matched cells from session 1 and session 2 (number of overlapping cells)/((number of session 1 cells + number of session 2 cells)/2).

The probability in Extended Data Fig. 7a–c was calculated by determining the probability of a subset of neurons from one session (for example, top 10% of Ctx A) to be present in the subset of neurons from a given percentile of activity in Ctx B (for example, top 20%)^17^. This was defined as *P*_A10,B20_ = *N*_A10,B20_/*U*, where *N*_A10,B20_ is the real number of neurons present on both percentiles and *U* is the universe of all cells detected in the concatenated video. The probability values were normalized by chance defined as *P*_A10_ × *P*_B20_ (0.1 × 0.2).

For statistical analyses of overlap significance in top 10% active neurons (Extended Data Fig. 7d), to evaluate whether the observed overlap of high-firing neurons between sessions exceeded chance levels, we performed a one-sided permutation test. For each subject, the top 10% most active neurons in Ctx A, HC1 and Ctx B were identified on the basis of descending firing rates. The real overlap between Ctx A and Ctx B or HC1 and Ctx B was computed as the proportion of top 10% neurons shared across the two sessions, excluding neurons present in the three sessions (that is, CtxA∩HC1∩CtxB). To establish a null distribution, we generated 1,000 shuffled datasets per subject by randomly permuting neuron identities in Ctx B while keeping the firing rate ranks in Ctx A and HC1 fixed. For each shuffle, we recalculated the overlap proportion. The *P* value was computed as the proportion of shuffled overlaps that were greater than or equal to the real (observed) overlap, reflecting the probability of obtaining such overlap by chance under the null hypothesis of no structured reactivation across sessions. Group-level *P* values were derived from aggregating real and shuffled overlap distributions across subjects.

### Whole-brain clearing

Brains were cleared according to the Adipo-clear protocol^56^, itself an adaption of the iDISCO protocol^57^. Animals were transcardially perfused as described above. Brains were then left overnight in 4% PFA at 4 °C. Hemisections of samples were taken by sagitally cutting the sample 2 mm to the right of the midline before being treated and immunostained^21^. Briefly, samples were dehydrated in a series of methanol washes before being washed in a dichloromethane solution. Samples were then bleached, rehydrated and incubated with primary antibody (rabbit anti-c-Fos; Synaptic Systems, 226008) at 1:500 for 11 days on a nutating orbital shaker at 37 °C. Following 2 days of washes, samples were then incubated with anti-rabbit Alexa Fluor 647 at 1:2,000 for 8 days at 37 °C. Samples were then washed for 3 days, dehydrated, washed in dichloromethane and finally cleared in dibenzyl ether.

### Whole-brain imaging

Brains were imaged using a light-sheet microscope (Ultramicroscope II, LaVision Biotec) that contained a ×2 (numerical aperture: 0.5) objective lens (MVPLAPO 2x), a dipping cap with a 6-mm working distance and a scientific CMOS camera (Andor Neo). Inspector Microscope v285 controller software was used to acquire images. All images were acquired at ×0.8 optical zoom. Samples were imaged with a step-size of 3 µm with a contrast adaptive algorithm for the 640-nm signal channel (set at 20 acquisitions per plane). The 488-nm channel was acquired without horizontal scanning.

### Whole-brain analysis

For model training, Light-sheet images of fluorescently labeled cell bodies were used to train a new model for c-Fos-positive detection, as described and implemented in the TrailMap pipeline^58^. Initial model weights were derived from a model previously trained on c-Fos-positive cells and further refined for this dataset. Three sessions of training with 20 steps per epoch and 100 epochs per session were performed. Each session included 8–12 hand-labeled image cubes (120 × 120 × 120 pixels). Training cubes were selected from different subjects across a variety of brain regions. Model performance was tested on new training cubes and compared to expert scoring.

For whole-brain c-Fos-positive quantification, image segmentation, registration and quantification were performed using the DeepCOUNT pipeline^59^. Briefly, the 488-nm autofluorescence channel was scaled and registered to the Gubra Lab light-sheet fluorescence microscopy atlas average template using annotations from the Allen CCF^60,61^. The same transformation was applied to the 640-nm channel. Transformed 640-nm images were processed using c-Fos-positive detection model in TrailMap. Model output probability maps were processed in MATLAB R2020b using the extended-maxima transform function and the connected component’s function. Count values per region are reported as the number of c-Fos-positive cells per volume of brain region in pixels.

### Statistics and reproducibility

Data collection and manual analysis were performed by experimenters blind to the experimental conditions. Sample sizes were based on prior studies from the laboratory and the field of study. Mice were pseudorandomly assigned to each group in all experiments. Control and experimental groups were matched by age and sex. Littermates were assigned to different groups whenever possible. All statistical analyses were performed using GraphPad Prism (version 10.2.3; GraphPad Software). In all figures, *n* designates the number of neurons and *N* designates the number of mice. Statistical significance was assessed using a Friedman test, multiple paired *t*-test with Holm–Šídák correction, one-way analysis of variance (ANOVA), two-way repeated measures (RM) ANOVA, Wilcoxon matched-pairs test, Mann–Whitney test or paired or unpaired Student’s *t*-test where appropriate, followed by the post hoc test indicated in figures. Normality was tested by Shapiro–Wilk and Kolmogorov–Smirnov tests. The level of significance was set at α < 0.05. Mice were excluded on the basis of an outlier test (Grubbs’ test 95% confidence) performed by GraphPad Prism software. A minimum of two experimental cohorts was used for Figs. 1e,f, 2b,c, 3c–h, 5a–l and 6a–f and Extended Data Figs. 1a,b, 6a–c and 7a–d. Representative histological images were repeated independently in different mice with similar results in Figs. 1c,d, 3b,i, 5d and 7e,m,r and Extended Data Figs. 4e, 5a–c, 9c and 10c,d.

### Reporting summary

Further information on research design is available in the Nature Portfolio Reporting Summary linked to this article.

## Online content

Any methods, additional references, Nature Portfolio reporting summaries, source data, extended data, supplementary information, acknowledgements, peer review information; details of author contributions and competing interests; and statements of data and code availability are available at 10.1038/s41593-026-02231-1.

## Supplementary information

Supplementary Information Supplementary Figs. 1–9 and Tables 1 and 2.

Reporting Summary

Supplementary Data 1 Supplementary Data 1: Source data for Supplementary Fig. 1.

Supplementary Data 2 Supplementary Data 2: Source data for Supplementary Fig. 2.

Supplementary Data 3 Supplementary Data 3: Source data for Supplementary Fig. 3.

Supplementary Data 4 Supplementary Data 4: Source data for Supplementary Fig. 4.

Supplementary Data 5 Supplementary Data 5: Source data for Supplementary Fig. 5.

Supplementary Data 6 Supplementary Data 6: Source data for Supplementary Fig. 6.

Supplementary Data 7 Supplementary Data 7: Source data for Supplementary Fig. 7.

Supplementary Data 8 Supplementary Data 8: Source data for Supplementary Fig. 8.

Supplementary Data 9 Supplementary Data 9: Source data for Supplementary Fig. 9.

## Source data

Source Data Fig. 1 Statistical source data.

Source Data Fig. 2 Statistical source data.

Source Data Fig. 3 Statistical source data.

Source Data Fig. 4 Statistical source data.

Source Data Fig. 5 Statistical source data.

Source Data Fig. 6 Statistical source data.

Source Data Fig. 7 Statistical source data.

Source Data Extended Data Fig. 1 Statistical source data.

Source Data Extended Data Fig. 2 Statistical source data.

Source Data Extended Data Fig. 3 Statistical source data.

Source Data Extended Data Fig. 4 Statistical source data.

Source Data Extended Data Fig. 5 Statistical source data.

Source Data Extended Data Fig. 6 Statistical source data.

Source Data Extended Data Fig. 7 Statistical source data.

Source Data Extended Data Fig. 8 Statistical source data.

Source Data Extended Data Fig. 9 Statistical source data.

Source Data Extended Data Fig. 10 Statistical source data.

## Data Availability

All raw data reported in this study are available from the corresponding authors upon request. We chose to share raw data upon request because of the large size of all the videos and images used in our analysis. Source data are provided with this paper.

## Code availability

Code for miniscope analysis (concat pipeline) can be found via Zenodo at 10.5281/zenodo.5676164 (ref. ^53^).
