# Supplementary material for: The prefrontal cortex controls memory organization in the hippocampus
Source: Nat Neurosci. 2026 Apr 28;29(5):1191–202. doi: 10.1038/s41593-026-02231-1 (PMC13156042; doi:10.1038/s41593-026-02231-1)
Supplement: Supplementary file 1 — Supplementary Figs. 1–9 and Tables 1 and 2. [file 41593_2026_2231_MOESM1_ESM.pdf]

nature neuroscience

Supplementary information https://doi.org/10.1038/s41593-026-02231-1

The prefrontal cortex controls memory organization in the hippocampus

In the format provided by the authors and unedited

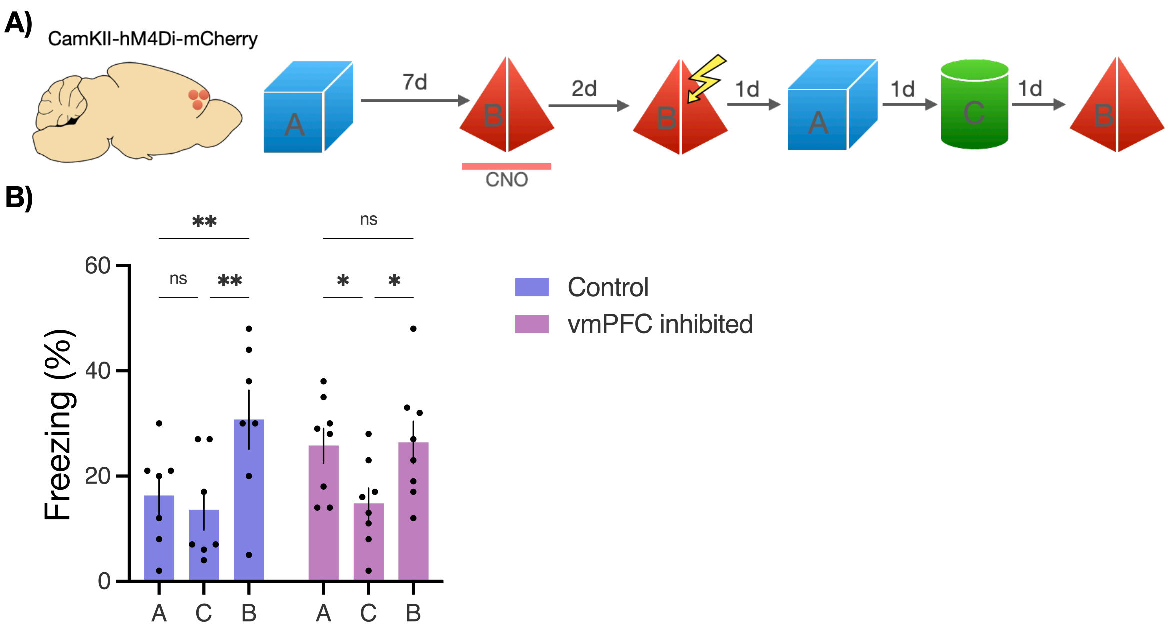

Supplementary Figure 1. Activity of vmPFC excitatory neurons controls context memory linking at 7 days. A) Schematic representation of the behavioral protocol used to test the role of vmPFC excitatory neurons in context memory linking. **B)** Freezing levels during exposure to context A, C or B following immediate shock in context B. Two-way RM ANOVA, two-tailed Tukey post-hoc test. Main effect of context: $F(2, 26)=11.47$, $p=0.003$, Control N=7, Inhibited N=8 per group). Bars represent mean ± SEM. *$p<0.05$, **$p<0.01$.

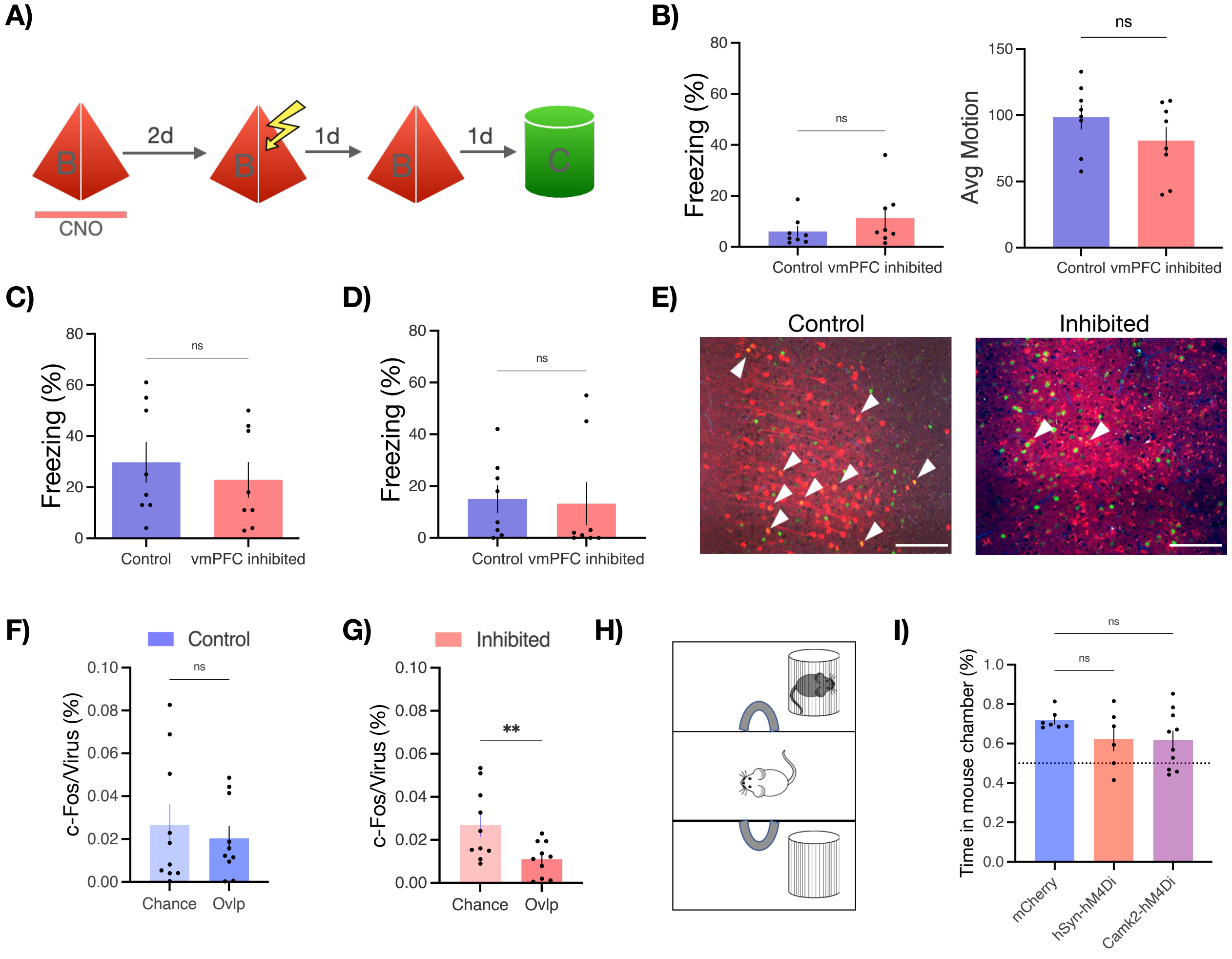

Supplementary Figure 2. Chemogenetic inhibition of vmPFC does not alter normal behavior. A) Schematic representation of the behavioral protocol used to test the impact of vmPFC inhibition on single memory encoding. **B)** Freezing levels and average motion index during memory encoding when vmPFC was inhibited. Two-tailed unpaired t-test. Freezing: t(14)=1.171, p=0.2613. Avg motion: t(14)=1.305, p=0.2129, N=8 per group). **C)** Freezing levels in context B one day following immediate shock. Two-tailed unpaired t-test t(14)=0.6621, p= 0.5187, N=8 per group. **D)** Freezing levels in a novel context C. Two-tailed unpaired t-test t(14)=0.1807, p=0.8592, N=8 per group. **E)** Representative image of cells expressing c-Fos protein (green) and viral proteins (red) in the control and inhibited groups. Arrows indicate overlapping cells. Scale bar: 100µm **F-G)** Percentage of overlapping cells compared to change levels in the control (E) and inhibited (F) groups. Two-tailed unpaired t-test. Control: t(9)=1.461, p=0.1780. Inhibited: t(9)=3.631, p=0.0055, N=10 per group. **H)** Schematic representation of the three-chamber assay used to evaluate the impact of vmPFC inhibition of social preference. I**)** Percentage of time spent on the mouse chamber. One-way ANOVA, two-tailed Dunnett's multiple comparison test. F(2, 20)=1.429, p=0.2631, mCherry N=7, hSyn-hM4Di N=6, Camk2 N=10. In all panels bars represent mean ± SEM. **p<0.01.

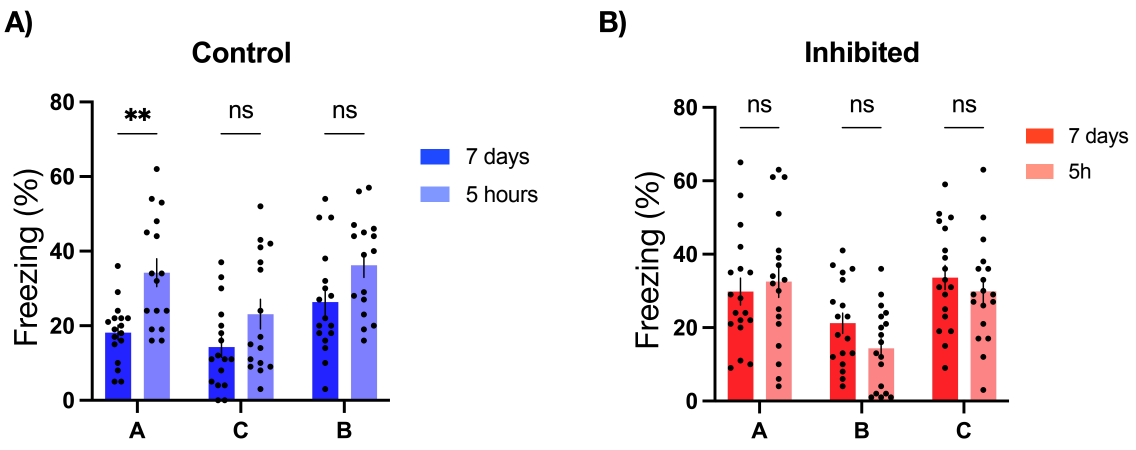

Supplementary Figure 3. Comparison of freezing levels at 5 hours and 7 days in control and vmPFC-inihbited groups. Related to figure 2. A) Comparison between mice in the 5h and 7d groups in controls. Two-way RM ANOVA, two-tailed Sidak's multiple comparisons test. Main effect of context $F(2, 62)=18.22$, $p<0.0001$. Main effect of group $F(1, 31) = 8.888$, $p=0.0055$, 7 days N=17, 5 hours N=16. **B)** Comparison between mice in the 5h and 7d groups in vmPFC inhibited. Two-way RM ANOVA, two-tailed Sidak's multiple comparisons test. Main effect of context $F(2, 68) = 27.33$, $p<0.0001$, 7 days N=18, 5 hours N=18. In all panels bars represent mean ± SEM. **$p<0.01$.

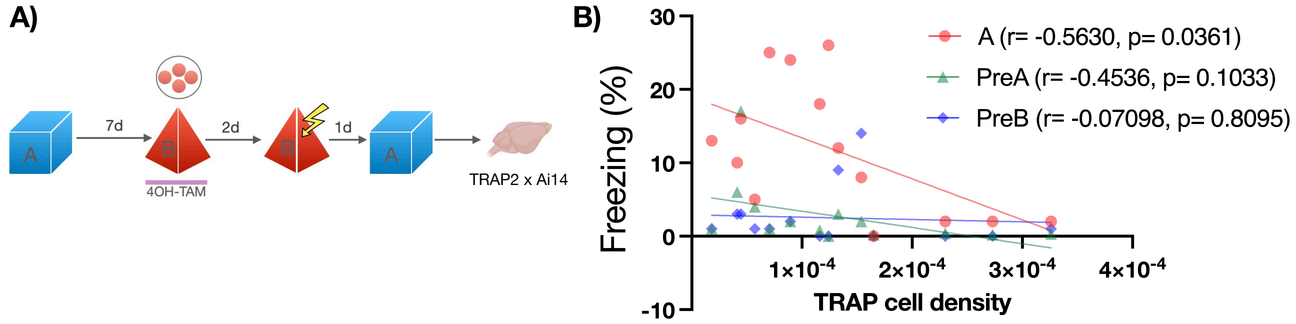

Supplementary Figure 4. Related to figure 2. The number of TRAP cells in the vmPFC correlates with freezing levels in context A after the shock, but not with freezing levels before the shock in contexts A or B. A) Schematic representation of the behavioral protocol used to evaluate the relationship between vmPFC activity and memory linking at 7-days in TRAP2xAi14 mice. **B)** Linear regression between freezing levels in context A after the shock (A), before the shock (PreA), and context B (PreB) and number of TRAP cells per ROI area (pixels) in the vmPFC. Pearson correlation coefficient is displayed for each regression, N=14 in all groups.

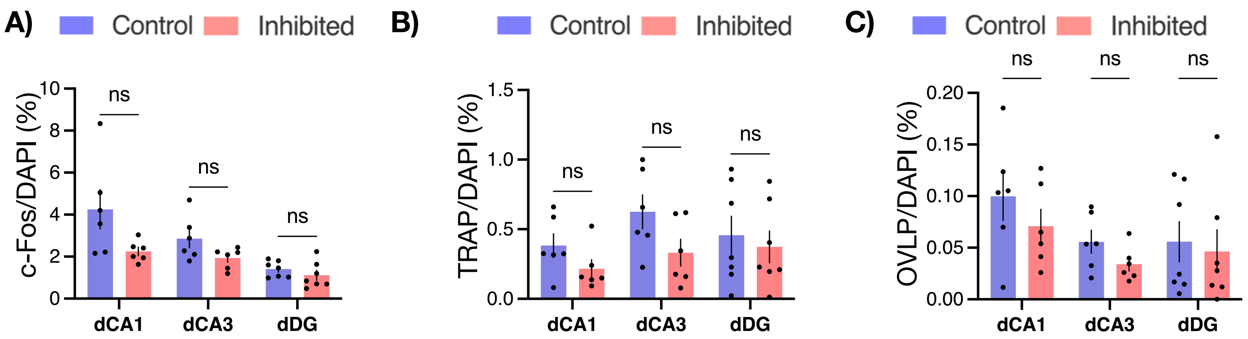

Supplementary Figure 5. Percentage of TRAP-, c-Fos-, and double-positive neurons on each subfield of the dHPC. Related to figure 3. Number of c-Fos-positive (A) , TRAP-positive cells (B) , or overlapping cells (C) divided by the number of DAPI-positive cells in each subfield. Multiple unpaired two-tailed t-test with Holm-Šídák correction. dCA1 N=6, dCA3 N=6, dDG N=7. In all panels bars represent mean ± SEM.

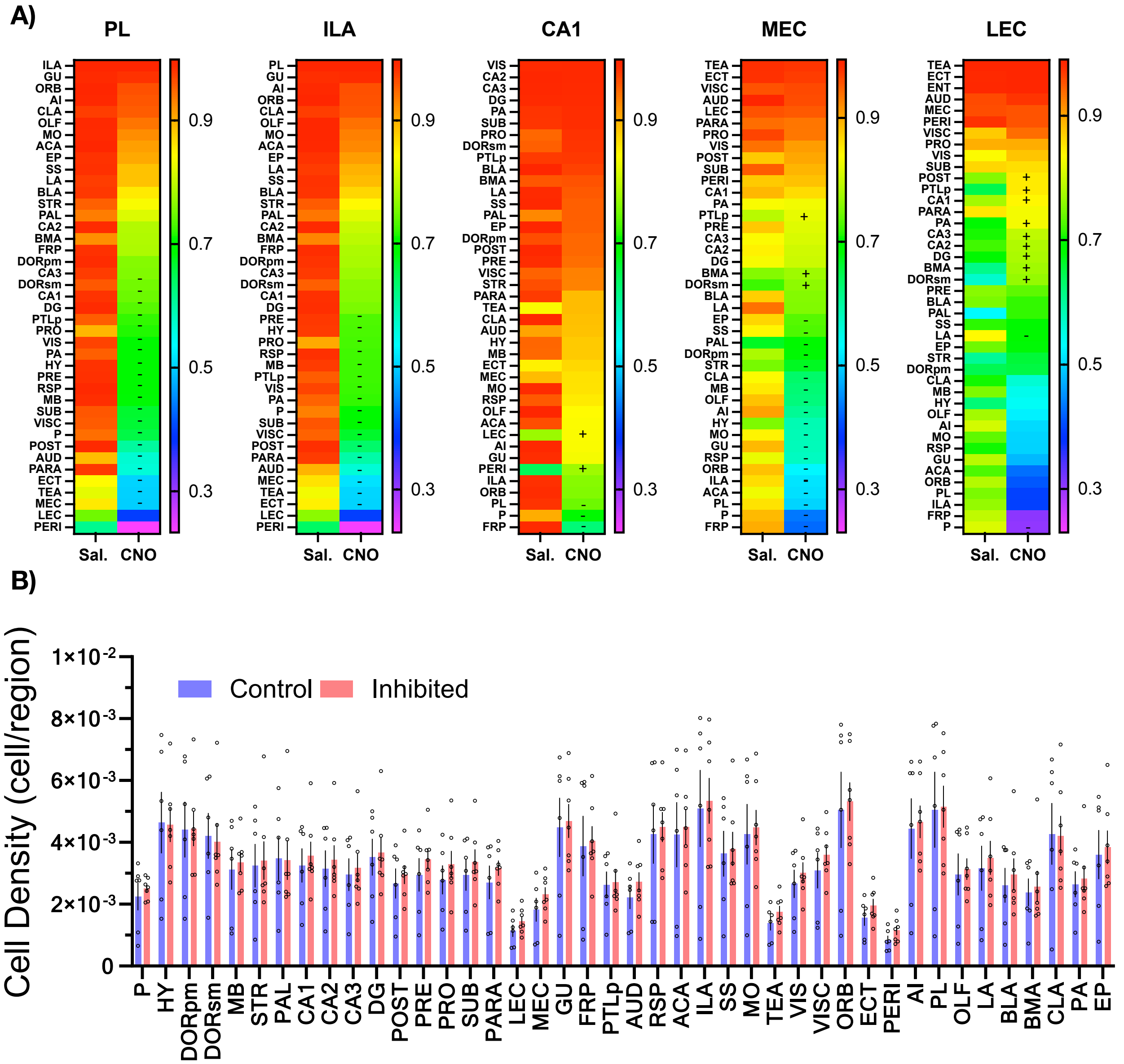

Supplementary Figure 6. Separate analysis of Entorhinal cortex areas (MEC and LEC). Related to figure 4. A) Pearson correlation coefficients sorted from highest to lowest in the inhibited group for HPC CA1, Prelimbic cortex (PL), Infralimbic cortex (ILA), Medial entorhinal cortex (MEC), and Lateral entorhinal cortex (LEC), Control N=6, Inhibited N=7. Overlayed "+" or "-" indicate regions that gained or lost a significant correlation upon vmPFC inhibition, respectively. **B)** Average number of c-Fos-positive cells per region volume (in pixels). Multiple two-tailed paired t-test with Holm-Šídák correction, Control N=6, Inhibited N=7. Bars represent mean ± SEM.

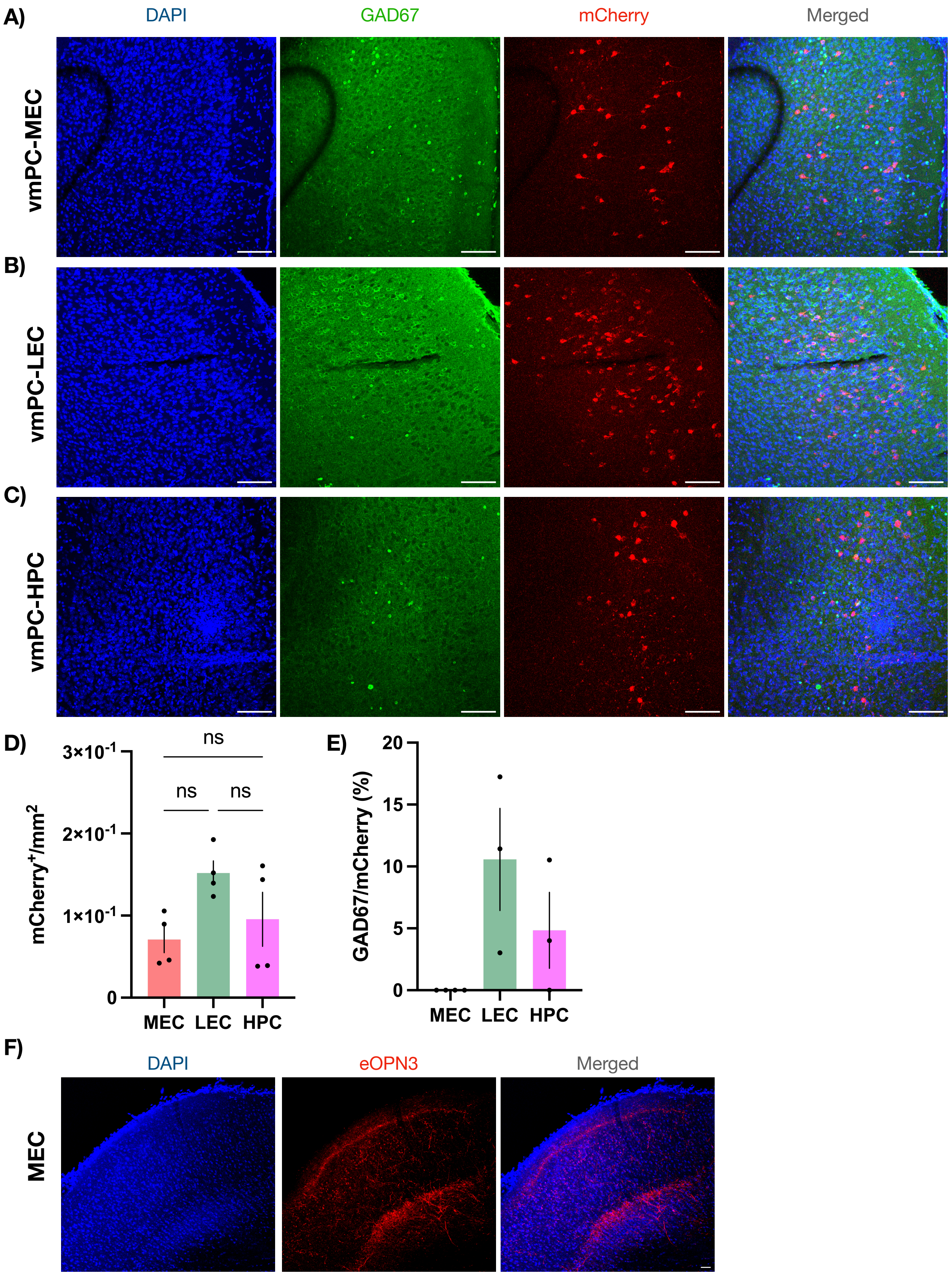
A)
DAPI
GAD67
mCherry
Merged
vmPC-MEC
B)
vmPC-LEC
C)
vmPC-HPC
D)
mCherry+/mm2
3×10-1
2×10-1
1×10-1
0
ns
ns
ns
MEC
LEC
HPC
E)
GAD67/mCherry (%)
20
15
10
5
0
MEC
LEC
HPC
F)
DAPI
eOPN3
Merged
MEC

Supplementary Figure 7. Characterization of vmPFC neurons projecting to different brain regions. A-C) Confocal image of vmPFC showing neurons that project to MEC, LEC, or HPC, identified by injecting RG-CRE in those regions and DIO-mCherry in the vmPFC (Red). Cells positive for the inhibitory neuronal marker GAD67 (Green) are also shown. **D)** Quantification of the number of mCherry-positive cells in the vmPFC that project to different regions. One-way ANOVA, two-tailed Tukey post-hoc test, $F(2, 9) = 3.329$, $p=0.0827$, $N=4$ per group. **E)** Quantification of the percentage of mCherry-positive cells that are also positive for GAD67 in the vmPFC. Individual points represent measures per slice, $n=3$-4 slices, $N=3$ mice. No statistical analysis was performed. **F)** Confocal image of the MEC showing vmPFC-MEC projections identified by injecting hSyn-CRE and SIO-eOPN3-mScarlet in the vmPFC. Scale bar: 100μm. In all panels, bars represent mean ± SEM.

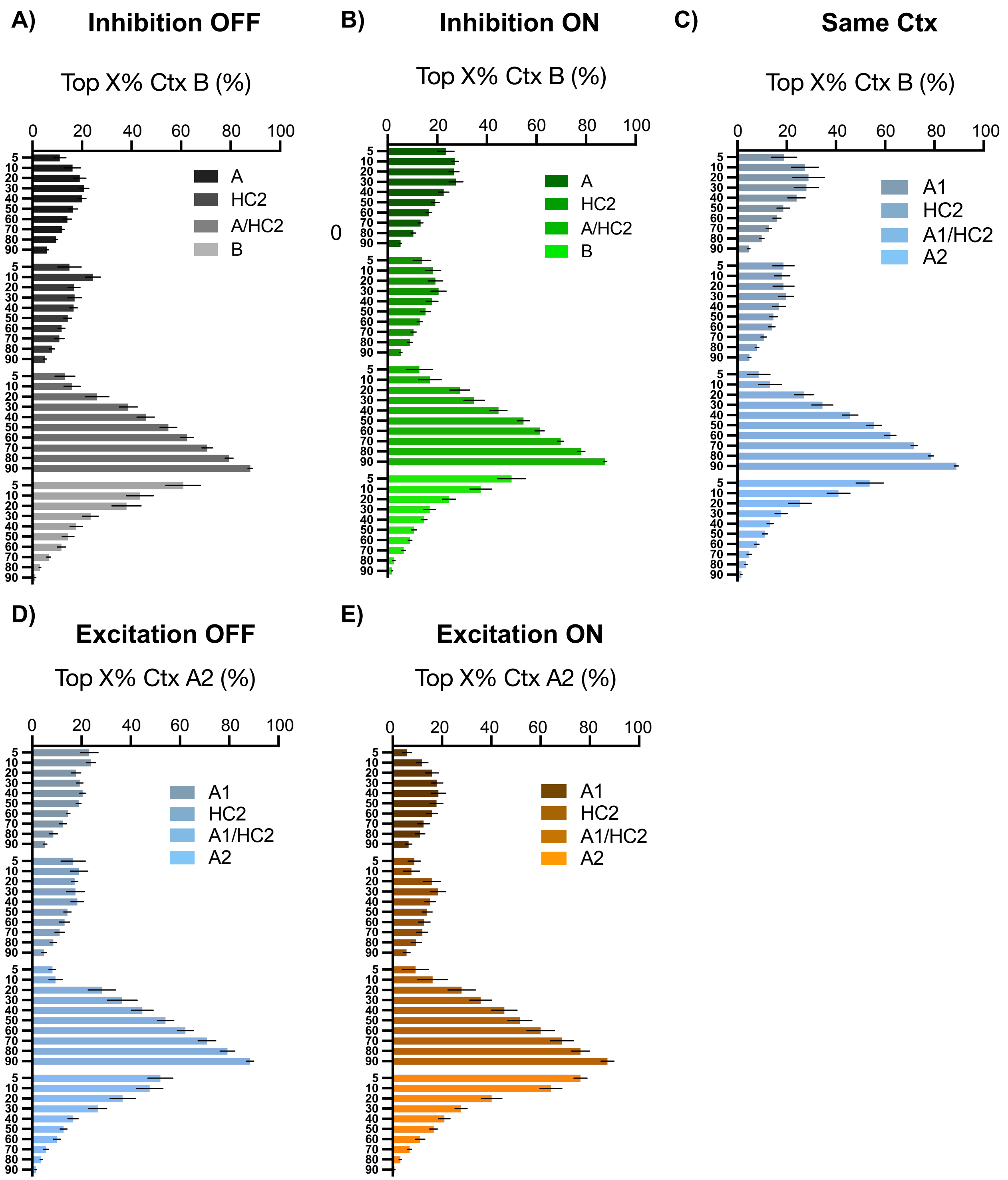

Supplementary Figure 8. Distribution analysis of the top X% active cells in each session as a percentage of the top X% top active cells in context B or A2. Related to figure 6. A-E) Percentage of neurons in the top X% most active cells in context B that are also in the top X% active cells in context A (A), home cage before the second context (HC2), both context A and HC2 (A/HC2), and exclusively active in the second context (B or A2) for different groups. A) N=11, B) N=11, C) N=11, D) N=5, E) N=5. In all panels bars represent mean ± SEM.

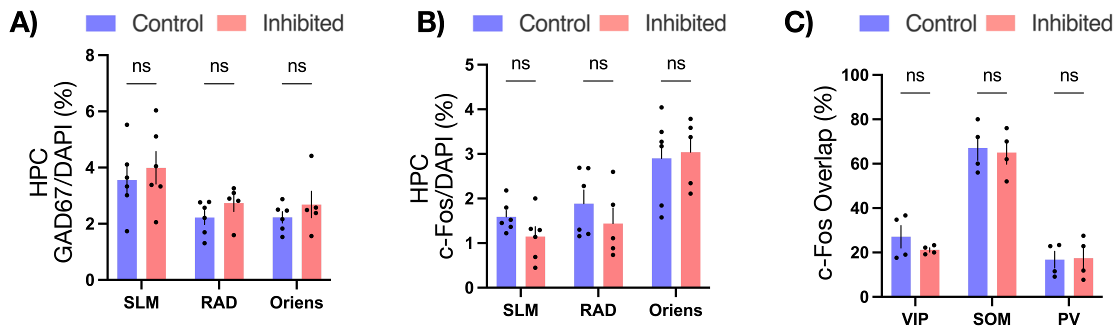

Supplementary Figure 9. Quantification of GAD67-positive, c-Fos-positive, and overlap with different inhibitory neurons in the HPC. Related to figure 7. A-B) Percentage of GAD67-positive (A) or c-Fos-positive (B) cells in different subfields of dCA1 with or without vmPFC-MEC inhibition. Multiple two-tailed unpaired t-test with Holm-Šídák correction. N=6. **C)** Percentage of c-Fos-positive cells for each class of inhibitory neurons in the dCA1 with or without vmPFC-MEC inhibition. Multiple two-tailed unpaired t-test with Holm-Šídák correction. N=4 per group. In all panels bars represent mean ± SEM.

Supplementary Table 1 and Table 2. Total number of cells used in the analyses of figure 6.

Table 1

Total Number of Cells in Inhibition Groups			
**Mouse #**	**Light OFF**	**Light ON**	**Same context (OFF)**
**6b**	174	173	218
**7b**	122	113	122
**5b**	146	165	143
**7a**	171	170	-
**8a**	59	98	94
**1b**	268	233	203
**4a**	26	40	39
**4b**	299	307	244
**2a**	74	113	34
**2b**	371	421	400
**3a**	242	161	191
**6a**	-	-	326

Table 2

Total Number of Cells in Excitation Groups		
**Mouse #**	**Light OFF**	**Light ON**
1	172	149
2	197	253
3	199	173
4	397	408
5	427	399
