# Supplementary material for: The prefrontal cortex controls memory organization in the hippocampus
Source: Nat Neurosci. 2026 Apr 28;29(5):1191–202. doi: 10.1038/s41593-026-02231-1 (PMC13156042; doi:10.1038/s41593-026-02231-1)
Supplement: Supplementary file 2 — Reporting Summary [file 41593_2026_2231_MOESM2_ESM.pdf]

nature portfolio

Corresponding author(s): André F. de Sousa; Alcino J. Silva

Last updated by author(s): Jul 30, 2024

Reporting Summary

Nature Portfolio wishes to improve the reproducibility of the work that we publish. This form provides structure for consistency and transparency in reporting. For further information on Nature Portfolio policies, see our Editorial Policies and the Editorial Policy Checklist.

Statistics

For all statistical analyses, confirm that the following items are present in the figure legend, table legend, main text, or Methods section.

n/a	Confirmed	
☐	☒	The exact sample size (*n*) for each experimental group/condition, given as a discrete number and unit of measurement
☐	☒	A statement on whether measurements were taken from distinct samples or whether the same sample was measured repeatedly
☐	☒	The statistical test(s) used AND whether they are one- or two-sided<br>*Only common tests should be described solely by name; describe more complex techniques in the Methods section.*
☒	☐	A description of all covariates tested
☐	☒	A description of any assumptions or corrections, such as tests of normality and adjustment for multiple comparisons
☐	☒	A full description of the statistical parameters including central tendency (e.g. means) or other basic estimates (e.g. regression coefficient) AND variation (e.g. standard deviation) or associated estimates of uncertainty (e.g. confidence intervals)
☐	☒	For null hypothesis testing, the test statistic (e.g. *F*, *t*, *r*) with confidence intervals, effect sizes, degrees of freedom and *P* value noted<br>*Give P values as exact values whenever suitable.*
☒	☐	For Bayesian analysis, information on the choice of priors and Markov chain Monte Carlo settings
☒	☐	For hierarchical and complex designs, identification of the appropriate level for tests and full reporting of outcomes
☐	☒	Estimates of effect sizes (e.g. Cohen's *d*, Pearson's *r*), indicating how they were calculated

Our web collection on statistics for biologists contains articles on many of the points above.

Software and code

Policy information about availability of computer code

Data collection	NIS-Elements AR Analysis software (Nikon, v.4.40.00); DAQ software (written in C++ and Open Computer Vision libraries for miniscope image acquisition (v0.171); Video Fear Conditioning "Video Freeze" Software (Med Associates); Imspector Microscope (v285)
Data analysis	NIS-Elements AR Analysis software (Nikon, v.4.40.00); Matlab R2020b (v9.9.01524771); GraphPad Prism, version 10.2.3 (347); TrailMap pipeline (2019 AlbertPun); DeepCOUNT 2019 pipeline; Python 3.7; ConcatMiniscope pipeline: Almeida-FilhoDG/ConcatMiniscope (vl.0.0, DOI: 10.5281/zenodo.5676164). Fear conditioning data was analyzed using Med Associates in built analysis.

For manuscripts utilizing custom algorithms or software that are central to the research but not yet described in published literature, software must be made available to editors and reviewers. We strongly encourage code deposition in a community repository (e.g. GitHub). See the Nature Portfolio guidelines for submitting code & software for further information.

Data

Policy information about availability of data

All manuscripts must include a data availability statement. This statement should provide the following information, where applicable:

- Accession codes, unique identifiers, or web links for publicly available datasets
- A description of any restrictions on data availability
- For clinical datasets or third party data, please ensure that the statement adheres to our policy

Source data for all figures are provided with this paper. All raw data reported in this study are available from the corresponding authors upon request. We chose to share raw data upon request due to the large size of all the videos and images used in our analysis.

Research involving human participants, their data, or biological material

Policy information about studies with human participants or human data. See also policy information about sex, gender (identity/presentation), and sexual orientation and race, ethnicity and racism.

Reporting on sex and gender	*Use the terms sex (biological attribute) and gender (shaped by social and cultural circumstances) carefully in order to avoid confusing both terms. Indicate if findings apply to only one sex or gender; describe whether sex and gender were considered in study design; whether sex and/or gender was determined based on self-reporting or assigned and methods used. Provide in the source data disaggregated sex and gender data, where this information has been collected, and if consent has been obtained for sharing of individual-level data; provide overall numbers in this Reporting Summary. Please state if this information has not been collected. Report sex- and gender-based analyses where performed, justify reasons for lack of sex- and gender-based analysis.*
Reporting on race, ethnicity, or other socially relevant groupings	*Please specify the socially constructed or socially relevant categorization variable(s) used in your manuscript and explain why they were used. Please note that such variables should not be used as proxies for other socially constructed/relevant variables (for example, race or ethnicity should not be used as a proxy for socioeconomic status). Provide clear definitions of the relevant terms used, how they were provided (by the participants/respondents, the researchers, or third parties), and the method(s) used to classify people into the different categories (e.g. self-report, census or administrative data, social media data, etc.) Please provide details about how you controlled for confounding variables in your analyses.*
Population characteristics	*Describe the covariate-relevant population characteristics of the human research participants (e.g. age, genotypic information, past and current diagnosis and treatment categories). If you filled out the behavioural & social sciences study design questions and have nothing to add here, write "See above."*
Recruitment	*Describe how participants were recruited. Outline any potential self-selection bias or other biases that may be present and how these are likely to impact results.*
Ethics oversight	*Identify the organization(s) that approved the study protocol.*

Note that full information on the approval of the study protocol must also be provided in the manuscript.

Field-specific reporting

Please select the one below that is the best fit for your research. If you are not sure, read the appropriate sections before making your selection.

☒ Life sciences ☐ Behavioural & social sciences ☐ Ecological, evolutionary & environmental sciences

For a reference copy of the document with all sections, see nature.com/documents/nr-reporting-summary-flat.pdf

Life sciences study design

All studies must disclose on these points even when the disclosure is negative.

Sample size	Sample size was determined based on prior studies in the field, including multiple studies from our lab that used the same behavioral paradigm, miniscope imaging and immunohistochemistry (Cai et al., 2016 PMID: 27251287; Shen etal., 2022 PMID: 35614219).
Data exclusions	Data were excluded based on outlier test (Grubbs' test 95% confidence) performed by Graphpad Prism software unless otherwise indicated.
Replication	Behavioral and miniscope experiments were repeated and results were reproducible. In addition, our main finding (increased overlap following vmPFC inhibition) was observed using 3 different techniques.For behavioral tasks, each result was collected from at least 2 independent experiments with similar results. Representative histological images were repeated independently in different mice (no less than 3) with similar results.
Randomization	Mice were pseudo-randomly assigned to each group in all experiments. Control and experiemntal groups were matched by age and sex. Litter mates were assigned to different groups whenever possible.
Blinding	Experimenter was blind during collection of behavioral data, with the exception of optogenetic experiments with light ON or OFF conditions.

Blinding	Experimenter was always blind to the groups during analysis of all experiments. Drug administration: experimenter was blinded to the vehicle/drug aliquot identity.

Reporting for specific materials, systems and methods

We require information from authors about some types of materials, experimental systems and methods used in many studies. Here, indicate whether each material, system or method listed is relevant to your study. If you are not sure if a list item applies to your research, read the appropriate section before selecting a response.

Materials & experimental systems

n/a	Involved in the study
☐	☒ Antibodies
☒	☐ Eukaryotic cell lines
☒	☐ Palaeontology and archaeology
☐	☒ Animals and other organisms
☒	☐ Clinical data
☒	☐ Dual use research of concern
☒	☐ Plants

Methods

n/a	Involved in the study
☒	☐ ChIP-seq
☒	☐ Flow cytometry
☒	☐ MRI-based neuroimaging

Antibodies

Antibodies used	Primary antibodies: rabbit anti-c-Fos (Cell Signaling, 9F6, #2250, 1:700); chicken anti-RFP (Synaptic Systems, #409006, 1:700); Mouse anti-GAD67, clone 1G10.2. (EMD Millipore, #MAB5406, 1:1000); rabbit anti-beta subunit Cholera Toxin antibody (Abcam, #ab34992, 1:1000); mouse anti-PV (Sigma-Aldrich, #P3088, 1:1000); guinea pig anti-VIP (Synaptic Systems, #443005, 1:100); rat anti-SOM (EMD Millipore, # MAB354, 1:100); guinea pig anti-Calbindin D28k (Synaptic Systems #214 004, 1:700); goat anti-Reelin polyclonal antibody (Invitrogen # PA5-47537, 1:1000).<br>Secondary antibodies: Goat anti-rabbit Alexa FluorTM 647 (Invitrogen, #A-21244, 1:1000); Goat anti-rabbit Alexa FluorTM 488 (Invitrogen, #A-11008, 1:1000); Goat anti-chicken Alexa FluorTM 594 (Invitrogen, #A-11042, 1:1000); Goat anti-mouse Alexa FluorTM 488 (Invitrogen, #A-11001, 1:1000); Goat anti-guinea pig Alexa FluorTM 568 (Invitrogen, #A-11075, 1:1000); Goat anti-Rat Alexa FluorTM 594 (Invitrogen, #A-11007, 1:1000); Donkey anti-goat Alexa FluorTM 647 (Invitrogen, #A-21447, 1:1000).
Validation	All antibodies were previously validated in other publications:<br>rabbit anti-c-Fos, Cell Signaling, 9F6, #2250: PMID: 40405277.<br>chicken anti-RFP, Synaptic Systems, #409006: PMID: 39962274.<br>Mouse anti-GAD67, clone 1G10.2. EMD Millipore, #MAB5406: PMID: 18980950.<br>rabbit anti-beta subunit Cholera Toxin antibody, Abcam, #ab34992: PMID: 40063795.<br>mouse anti-PV, Sigma-Aldrich, #P3088:PMID: 19892435.<br>guinea pig anti-VIP, Synaptic Systems, #443005: PMID: 39738072.<br>rat anti-SOM, EMD Millipore, # MAB354: PMID: 25787832.<br>guinea pig anti-Calbindin D28k, Synaptic Systems #214 004: PMID: 41288860.<br>goat anti-Reelin polyclonal antibody, Invitrogen # PA5-47537: PMID: 36468693.

Animals and other research organisms

Policy information about studies involving animals; ARRIVE guidelines recommended for reporting animal research, and Sex and Gender in Research

Laboratory animals	Mus musculus, male and female 12-24 week-old, (1) C57BL/6NTac, Taconic Farms, (2) Fos2A-iCreER (TRAP2) (RRID:IMSR_JAX:030323, Jackson Laboratory), (3) R26AI14/+ (AI14) (RRID:IMSR_JAX:007914, Jackson Laboratory), (3) Ndnftm1.1(cre)Rudy/J (NDNF-CRE) (RRID:IMSR_JAX:030757, Jackson Laboratory). Transgenic mice were maintained on a C57BL/6 Jackson background.<br>All mice used in this study were housed in AAALAC accredited facility with free access to food and water, and maintained on a 12:12 hour light: dark cycle. All studies were approved by the Chancellor's Animal Research Committee at UCLA. Housing conforms to The Guide for Care and USe of Laboratory Animals, 8th ed. Temperature setpoint was 72 degrees Fahrenheit and humidity between 30-70%
Wild animals	NA
Reporting on sex	Both male and female mice were used. No sex-specific analysis was performed.
Field-collected samples	NA
Ethics oversight	All studies were approved by the Chancellor's Animal Research Committee at UCLA.

Note that full information on the approval of the study protocol must also be provided in the manuscript.

Plants

Seed stocks	*Report on the source of all seed stocks or other plant material used. If applicable, state the seed stock centre and catalogue number. If plant specimens were collected from the field, describe the collection location, date and sampling procedures.*
Novel plant genotypes	*Describe the methods by which all novel plant genotypes were produced. This includes those generated by transgenic approaches, gene editing, chemical/radiation-based mutagenesis and hybridization. For transgenic lines, describe the transformation method, the number of independent lines analyzed and the generation upon which experiments were performed. For gene-edited lines, describe the editor used, the endogenous sequence targeted for editing, the targeting guide RNA sequence (if applicable) and how the editor was applied.*
Authentication	*Describe any authentication procedures for each seed stock used or novel genotype generated. Describe any experiments used to assess the effect of a mutation and, where applicable, how potential secondary effects (e.g. second site T-DNA insertions, mosiacism, off-target gene editing) were examined.*
